# Clinical implications of monitoring nivolumab immunokinetics in non–small cell lung cancer patients

**DOI:** 10.1172/jci.insight.59125

**Published:** 2018-10-04

**Authors:** Akio Osa, Takeshi Uenami, Shohei Koyama, Kosuke Fujimoto, Daisuke Okuzaki, Takayuki Takimoto, Haruhiko Hirata, Yukihiro Yano, Soichiro Yokota, Yuhei Kinehara, Yujiro Naito, Tomoyuki Otsuka, Masaki Kanazu, Muneyoshi Kuroyama, Masanari Hamaguchi, Taro Koba, Yu Futami, Mikako Ishijima, Yasuhiko Suga, Yuki Akazawa, Hirotomo Machiyama, Kota Iwahori, Hyota Takamatsu, Izumi Nagatomo, Yoshito Takeda, Hiroshi Kida, Esra A. Akbay, Peter S. Hammerman, Kwok-kin Wong, Glenn Dranoff, Masahide Mori, Takashi Kijima, Atsushi Kumanogoh

**Affiliations:** 1 Department of Respiratory Medicine and Clinical Immunology, Osaka University Graduate School of Medicine, Suita, Osaka, Japan.; 2 Laboratory of Immunopathology, WPI Immunology Frontier Research Center, Osaka University, Suita, Osaka, Japan.; 3 Department of Thoracic Oncology, National Hospital Organization, Toneyama National Hospital, Toyonaka, Osaka, Japan.; 4 Department of Immunology and Genomics, Osaka City University Graduate School of Medicine, Osaka, Osaka, Japan.; 5 Division of Innate Immune Regulation, International Research and Development Center for Mucosal Vaccines, Institute of Medical Science, The University of Tokyo, Tokyo, Japan.; 6 DNA-Chip Developmental Center for Infectious Diseases, Research Institute for Microbial Diseases, Osaka University, Suita, Osaka, Japan.; 7 Department of Pathology, University of Texas Southwestern Medical Center at Dallas, Dallas, Texas, USA.; 8 Novartis Institutes for Biomedical Research, Cambridge, Massachusetts, USA.; 9 Laura and Isaac Perlmutter Cancer Center, New York University Langone Medical Center, New York, New York, USA.; 10 Integrated Frontier Research for Medical Science Division, Institute for Open and Transdisciplinary Research Initiatives, Osaka University, Suita, Osaka, Japan.

**Keywords:** Immunology, Pulmonology, Cancer immunotherapy, Lung cancer

## Abstract

**BACKGROUND.** The PD-1–blocking antibody nivolumab persists in patients several weeks after the last infusion. However, no study has systematically evaluated the maximum duration that the antibody persists on T cells or the association between this duration and residual therapeutic efficacy or potential adverse events.

**METHODS.** To define the duration of binding and residual efficacy of nivolumab after discontinuation, we developed a simplified strategy for T cell monitoring and used it to analyze T cells from peripheral blood from 11 non–small cell lung cancer patients previously treated with nivolumab. To determine the suitability of our method for other applications, we compared transcriptome profiles between nivolumab-bound and nivolumab-unbound CD8 T cells. We also applied T cell monitoring in 2 nivolumab-treated patients who developed progressive lung tumors during long-term follow-up.

**RESULTS.** Prolonged nivolumab binding was detected more than 20 weeks after the last infusion, regardless of the total number of nivolumab infusions (2–15 doses) or type of subsequent treatment, in 9 of the 11 cases in which long-term monitoring was possible. Ki-67 positivity, a proliferation marker, in T cells decreased in patients with progressive disease. Transcriptome profiling identified the signals regulating activation of nivolumab-bound T cells, which may contribute to nivolumab resistance. In 2 patients who restarted nivolumab, T cell proliferation markers exhibited the opposite trend and correlated with clinical response.

**CONCLUSIONS.** Although only a few samples were analyzed, our strategy of monitoring both nivolumab binding and Ki-67 in T cells might help determine residual efficacy under various types of concurrent or subsequent treatment.

**TRIAL REGISTRATION.** University Hospital Medical Information Network Clinical Trials Registry, UMIN000024623.

**FUNDING.** This work was supported by Japan Society for the Promotion of Science KAKENHI (JP17K16045, JP18H05282, and JP15K09220), Japan Agency for Medical Research and Development (JP17cm0106310, JP18cm0106335 and JP18cm059042), and Core Research for Evolutional Science and Technology (JPMJCR16G2).

## Abstract 2

A method for detecting nivolumab binding and T cell activation status, which could be used to predict residual efficacy and toxicity, is developed.

## Introduction

Recent clinical trials demonstrated that a specific subset of non–small cell lung cancer (NSCLC) patients exhibit a clear response to PD-1 blockade (1–3) and that the benefit of this treatment is greater than that of platinum doublet chemotherapy when the level of PD-L1 expression in tumors is higher (4). Although selection of the high PD-L1 expression subset enriches the response to anti–PD-1 therapy (5), more than 50% of cases still do not exhibit durable responses to PD-1 blockade. For these patients, sequential therapeutic regimens are required. In addition, immunotherapy-specific immune-related adverse events (irAEs), which potentially affect almost all tissues and can occur long after treatment, are also of concern when using PD-1 blockade (6, 7). On the other hand, a subset of responders to PD-1 blockade present with a long-term clinical response even after discontinuation of the therapy (8). These issues highlight the importance of monitoring the kinetics of PD-1–blocking antibodies after discontinuation, in order to predict residual efficacy of PD-1 blockade and manage the symptoms of irAEs.

A method for monitoring the occupancy of nivolumab, an anti–PD-1 antibody, on T cells was originally reported as a part of clinical trials, and therapeutic antibody binding was confirmed up to day 85 (9, 10). However, it remains to be fully investigated how subsequent therapeutic regimens, including corticosteroids, affect nivolumab binding after discontinuation and whether prolonged nivolumab activity, loss of activity, or irAEs can be monitored based on nivolumab-binding status. It is also unclear whether plasma concentrations of nivolumab correlate well with efficacy or with residual nivolumab binding to T cells.

Here, we developed a simplified strategy to monitor both sustained binding of nivolumab and occupancy of PD-1 on T cells by nivolumab. In this approach, we performed flow cytometry of freshly collected whole blood or pleural effusion samples from NSCLC patients who received nivolumab as part of their treatment. This approach is a modification of a previously reported method (9, 11). Our method uses less than 100 μl whole blood and does not require a mononuclear cell enrichment step. Our monitoring technique revealed that nivolumab-binding status on T cells can be classified into 3 different groups: complete binding (CB), partial binding (PB), and no binding (NB). Although T cells with CB never interact with PD-L1, T cells with PB can potentially be inhibited by PD-L1. CB status in T cells from peripheral blood lasted at least 19 weeks, regardless of the duration of nivolumab treatment and the content of subsequent therapy, in 9 patients who were monitored over the long term. PB status in these samples was confirmed after 25–43 weeks. We also evaluated the concentration of nivolumab in plasma by ELISA. The long-term persistence of residual binding was due to the sustained presence of free nivolumab in plasma. However, the plasma concentration of nivolumab differed among individual patients at the time of absolute loss of CB, probably resulting from the variations in total counts of PD-1^+^ cells or differences in drug pharmacokinetics among patients. Along with the binding status of nivolumab, we also monitored the status of differentiation markers (CD45RA, CCR7, and Tbet) and proliferation status (Ki-67) in T cells, because it is well established that these markers reflect T cell reinvigoration induced by PD-1–blocking antibodies (11–14). Although the differentiation markers did not exhibit any specific trend during long-term follow-up, Ki-67 positivity in both nivolumab-bound and total T cells decreased in patients whose tumors did not respond to subsequent treatments. In particular, Ki-67 positivity was significantly reduced at the time of disease progression, although the number of patients used to reach this conclusion was limited. In addition, we developed a strategy for specifically sorting the nivolumab-bound T cell population in peripheral blood and subjected RNA from these enriched nivolumab-bound T cell samples to transcriptome analysis. Pathway analysis suggested that regulatory signals of T cell activation were upregulated in nivolumab-bound CD8 T cells relative to nivolumab-unbound CD8 T cells. Finally, we monitored nivolumab binding and Ki-67 positivity in T cells from two clinical cases previously treated with nivolumab. In both patients, the tumors initially responded to nivolumab, but treatment was discontinued due to irAEs (myositis in one case and thrombocytopenia in the other). Because the severity of irAEs was mild, nivolumab treatment was cautiously restarted. Nivolumab binding was confirmed in both cases; however, Ki-67 positivity exhibited the opposite trend, correlating with the clinical response to reinitiation of this treatment.

Although a large-scale prospective trial will be necessary to draw definitive conclusions, our monitoring strategy was feasible in a real-world cohort. Moreover, combinatorial monitoring of both anti–PD-1 therapeutic antibody binding and Ki-67 positivity in T cells could be used to predict residual efficacy and toxicity of PD-1 blockade, even in the face of systemic chemotherapy and radiation treatment.

## Results

### A strategy for detecting nivolumab-binding status on T cells.

**Figure 1:**
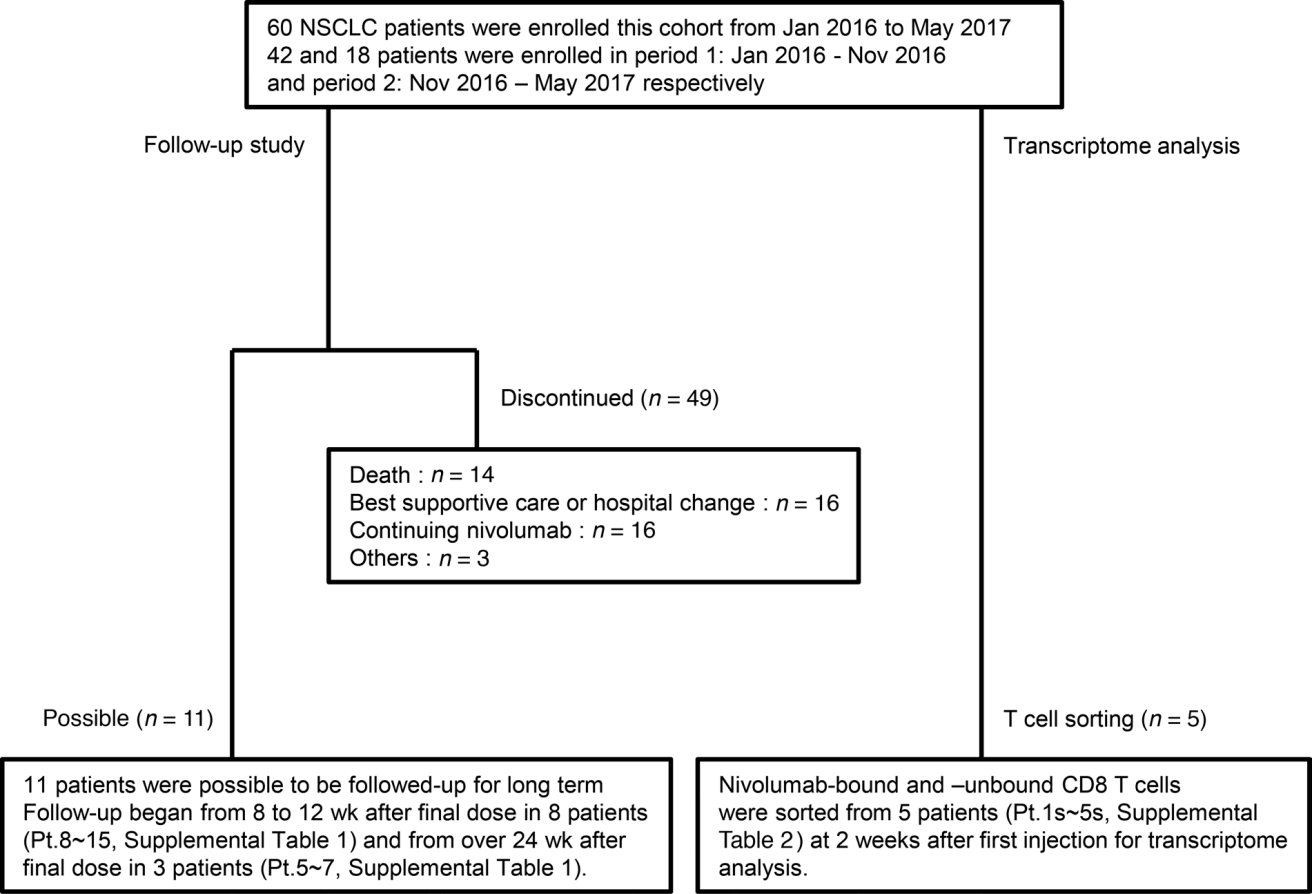
Diagram of patient disposition. A total of 60 non–small cell lung cancer (NSCLC) patients, who started nivolumab treatment as second-line or further-line treatment from January 2016 to May 2017 were enrolled in this cohort. Of those, 11 could be followed up over 8 weeks after nivolumab discontinuation, and ultimately 9 patients were followed-up over the long term. Follow-up study design and schedules are presented in Supplemental Table 1. Five patients from period 2 were selected as representatives for transcriptome analysis.

We initiated this study to prospectively profile immune cells from freshly isolated peripheral blood from NSCLC patients using flow cytometry. A total of 60 NSCLC patients were enrolled in this study and received nivolumab treatment as second-line or further-line treatment at Osaka University Hospital or Toneyama National Hospital between January 2016 and May 2017 (Figure 1 and Supplemental Tables 1 and 2; supplemental material available online with this article; https://doi.org/10.1172/jci.insight.59125DS1).

**Figure 2:**
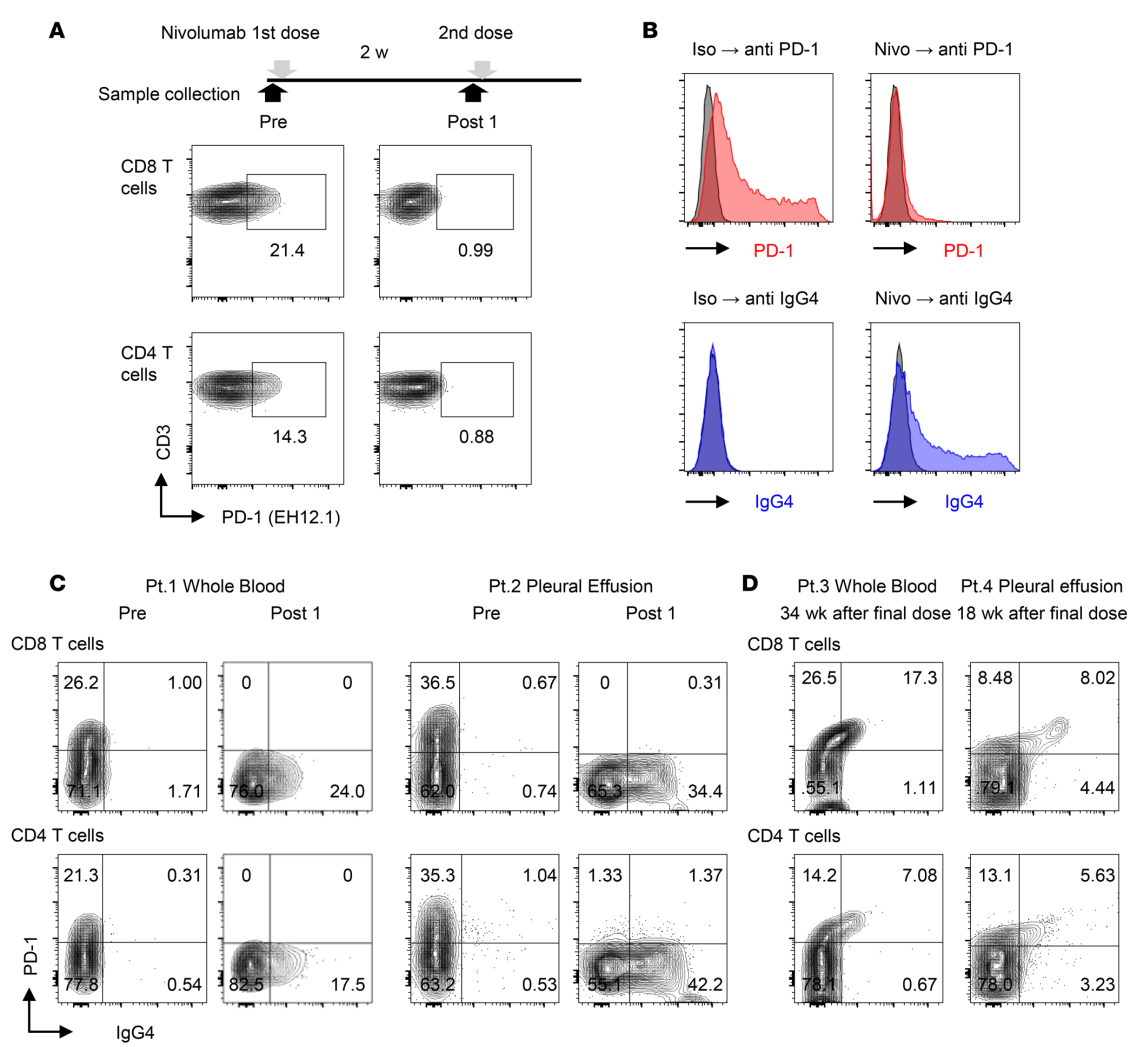
A method for detection of nivolumab binding in T cells of NSCLC patients. (**A**) Flow cytometry staining of PD-1 with PD-1 detection antibody (EH12.1) in peripheral blood CD8 and CD4 T cells before injection (Pre) and 2 weeks after the first dose of nivolumab (Post 1). (**B**) PD-1–transfected HEK293T cells (HEK293T-PD-1) exhibited binding of EH12.1 (left). After treatment with nivolumab, EH12.1 binding was completely abolished, whereas the nivolumab detection antibody (anti-IgG4, HP6025) exhibited the original expression pattern (right). Data are representative of 3 independent experiments. (**C**) Staining of PD-1 by EH12.1 and IgG4 by HP6025 in CD8 and CD4 T cells from fresh whole blood and pleural effusion was evaluated by flow cytometry at pretreatment and 2 weeks after the initial dose of nivolumab. Whole blood from Pt. 1 and pleural effusion from Pt. 2 are shown as representative analyses. (**D**) Staining of PD-1 (EH12.1) and IgG4 (HP6025) in CD8 and CD4 T cells from fresh whole blood from Pt. 3 and pleural effusion from Pt. 4 were analyzed by flow cytometry at 34 weeks and 18 weeks after the final dose, respectively.

To detect PD-1 expression on T cells, we stained the cells using an anti–PD-1 antibody, EH12.1. This antibody could detect PD-1 before nivolumab treatment, but detection was lost after nivolumab infusion (Figure 2A). To determine whether nivolumab binding to T cells hinders detection of PD-1 by EH12.1, we overexpressed PD-1 in HEK293T cells (HEK293T-PD-1) and evaluated competitive binding between EH12.1 and nivolumab. The results confirmed that nivolumab treatment inhibited recognition by EH12.1. Next, we used an anti-IgG4 antibody, HP6025, to detect nivolumab binding (Figure 2B). Utilizing these antibodies, we observed a switch in the staining pattern from PD-1^+^ to IgG4^+^ in T cells from blood and pleural effusion from NSCLC patients (patient [Pt.] 1 and Pt. 2) after a single dose of nivolumab (Figure 2C). All characteristics of NSCLC patients analyzed in this study are provided in Supplemental Table 2.

To evaluate how the PD-1^+^ and IgG4^+^ staining pattern in T cells changes long after discontinuation of treatment, we examined peripheral blood at 34 weeks after the last infusion (Pt. 3) and pleural effusion at 18 weeks after the last infusion (Pt. 4). At these time points, T cells were predominantly PD-1^+^IgG4^+^ (Figure 2D), in contrast to the PD-1^–^IgG4^+^ pattern detected in patients treated with a single dose and analyzed 2 weeks afterward (Figure 2C). This suggests that the staining pattern reflects the nivolumab saturation status of PD-1 on T cells.

**Figure 3:**
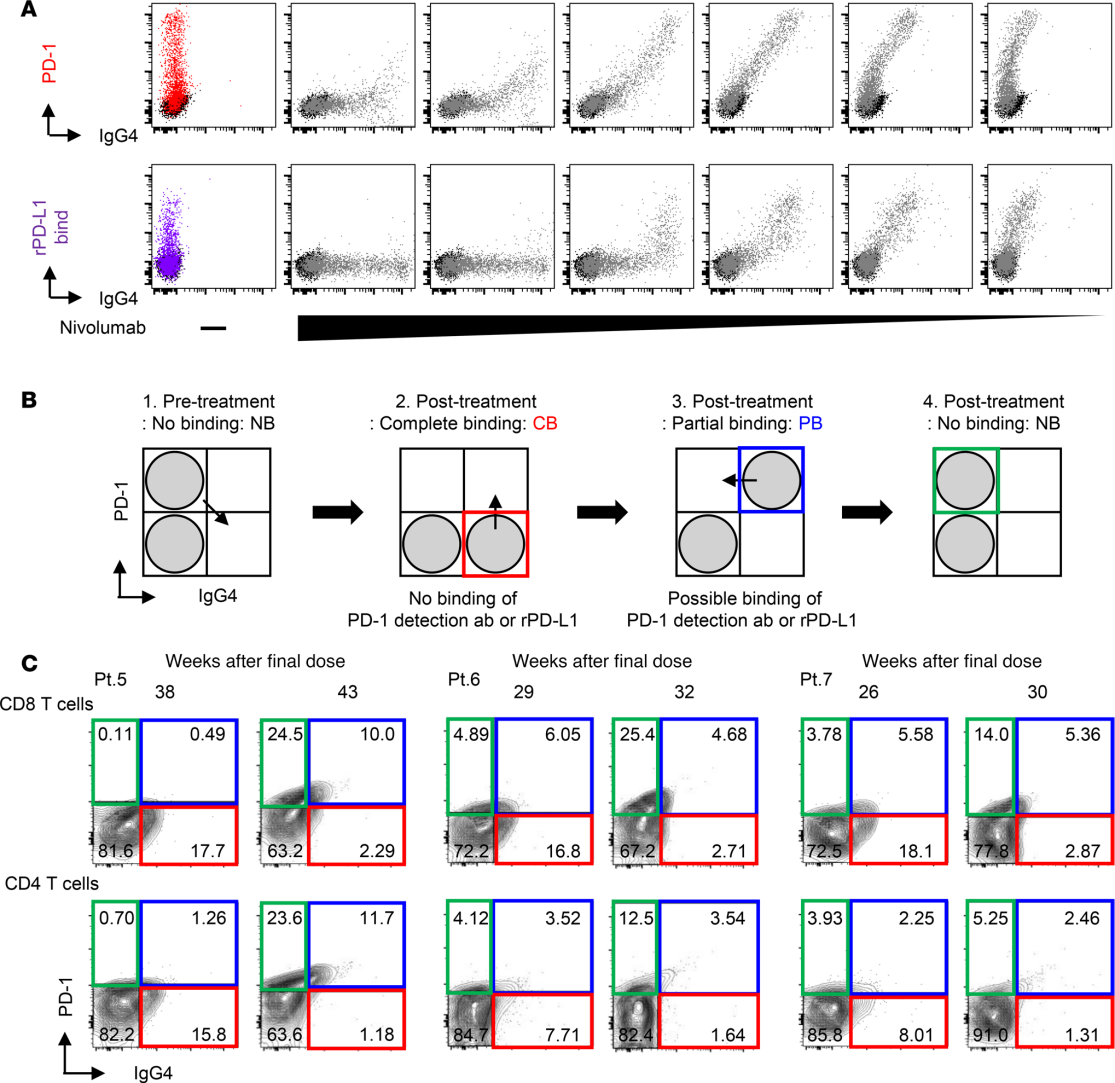
The change in nivolumab-binding status can be mapped by flow cytometry. (**A**) Human PD-1–transfected HEK293T cells (HEK293T-PD-1) were treated with serial dilutions of nivolumab (10, 2, 0.4, 0.08, 0.016, and 0.0032 μg/ml), and the staining patterns of IgG4 and PD-1 detection antibodies and the capacity of recombinant PD-L1 binding (20 μg/ml) were evaluated. Data are representative of 3 independent experiments. (**B**) Schematics present 3 different binding statuses: complete binding (CB: red gate), partial binding (PB: blue gate), and no binding (NB: green gate). (**C**) Nivolumab binding was analyzed at 2 follow-up time points, as indicated, in fresh peripheral blood CD8 and CD4 T cells from each of 3 cases that discontinued treatment (Pt. 5–7).

To assess whether the PD-1^+^IgG4^+^ pattern is proportional to the occupancy of PD-1, we treated HEK293T-PD-1 cells with serially diluted concentrations of nivolumab and then evaluated the binding capacity of recombinant PD-L1 in a nivolumab dose-dependent manner. We found that the staining pattern in PD-1 versus IgG4 antibodies correlated well with the ability of recombinant PD-L1 to bind unoccupied PD-1 on T cells (Figure 3A), suggesting that our flow cytometry data reflect the true saturation status of PD-1 on T cells. Based on the staining pattern, we concluded that there are 3 patterns: CB (PD-1^–^IgG4^+^ subset), PB (PD-1^+^IgG4^+^ subset), and NB (PD-1^+^IgG4^–^ subset) (Figure 3B).

During period 1 (Figure 1 and Supplemental Table 1), we completed the optimization of the method for evaluating the binding of nivolumab to T cells, as described. In November 2016, we initiated the follow-up monitoring study, in which 11 of 60 patients were enrolled (Supplemental Table 1). To confirm that these binding patterns can also be monitored in peripheral blood CD8 T and CD4 T cells from NSCLC patients, we compared the nivolumab-binding status of 6 patients at 2 different time points. The data from 3 patients who discontinued nivolumab treatment (Pt. 5–7) and 3 representative patients who continued nivolumab treatment are shown in Figure 3C and Supplemental Figure 1, respectively. The percentage of CB was consistent in all 3 patients who continued nivolumab (Supplemental Figure 1), whereas the percentage of CB decreased and the percentage of PB plus NB increased at 2 follow-up points, as indicated, after nivolumab discontinuation in all 3 patients (Figure 3C).

### Nivolumab binding on memory T cells in blood is detectable more than 20 weeks after the final dose, regardless of the number of nivolumab doses and the content of subsequent treatment.

**Figure 4:**
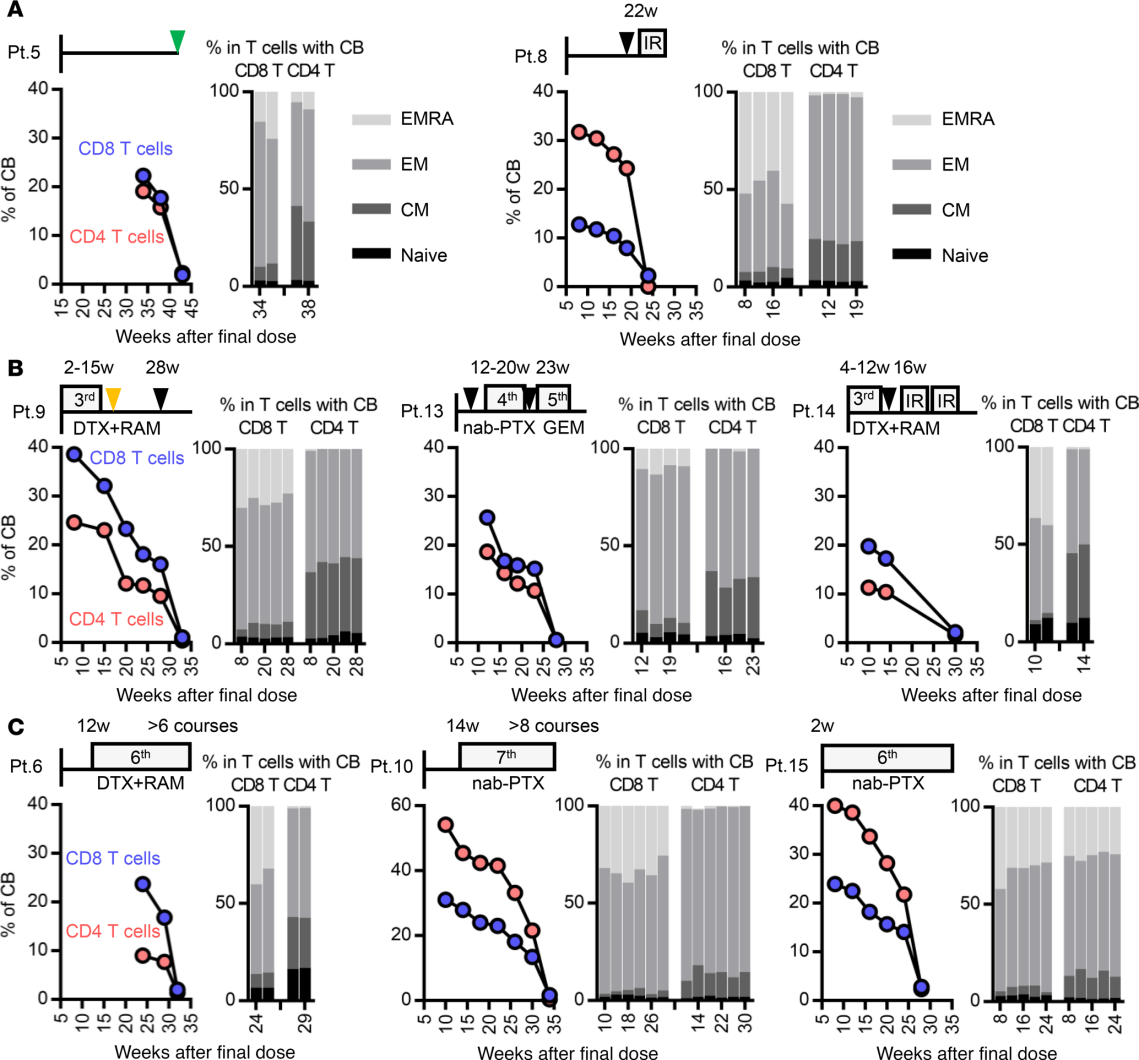
Following up binding status and differentiation markers in nivolumab-bound T cells in patients who underwent sequential treatment. Fresh whole blood samples from 8 non–small cell lung cancer patients were followed up in terms of percentage of complete binding of nivolumab (left) and the ratio of 4 subsets (CD45RA^+^CCR7^+^ naive, CD45RA^–^CCR7^+^ central memory [CM], CD45RA^–^CCR7^–^ effector memory [EM], and CD45RA^+^CCR7^–^ effector memory RA [EMRA]) in the CB population (right) of CD8 and CD4 T cells. (**A**) Pt. 5 and 8 discontinued nivolumab treatment due to irAE. (**B**) Pt. 9, 13, and 14 and (**C**) Pt. 6, 10, and 15 discontinued nivolumab treatment due to progressive disease. (**C**) Pt. 6, 10, and 15 were well controlled by subsequent chemotherapies for more than 24 weeks. Black and green triangles indicate the points of progressive disease and tumor marker re-elevation without an increase in the size of the targeted tumor (as determined by CT scan), respectively. The orange triangle indicates the point of an adverse event of chemotherapy.

We applied our method to evaluate nivolumab-binding status and detect additional T cell markers for long-term monitoring in NSCLC patients. Eleven patients (Pt. 5–15) were followed up every 4 ± 1 weeks at times more than 8 weeks after the final injection of nivolumab, until CB was lost. Follow-up had to be discontinued for Pt. 11 and Pt. 12 (Supplemental Table 1). We performed long-term follow-up for the remaining 9 patients who discontinued nivolumab treatment. Pt. 5 exhibited mild thrombocytopenia around 10 weeks after initiation of nivolumab. Although the magnitude of thrombocytopenia did not reach grade 1 (CTCAE version 4.0), thrombocytopenia became more severe at 22 weeks with increased titer of PA-IgG. Therefore, we ceased nivolumab at that time point (Figure 4A, left). Pt. 8 presented with grade 2 dermatitis around 16 weeks after the initiation of nivolumab. At the beginning, nivolumab was continued cautiously in conjunction with topical corticosteroids. However, the dermatitis did not response well to this treatment, and the patient developed a fever 26 weeks after the initiation of nivolumab; consequently, nivolumab was discontinued at the time point (Figure 4A, right). In Pt. 6, 7, 9, 10, 13, 14, and 15, nivolumab was discontinued due to progressive disease (PD) (Figure 4, B and C, and Supplemental Figure 2). The latter 7 cases received subsequent chemotherapy, including docetaxel (DTX) plus ramucirumab (RAM), nab-paclitaxel (nab-PTX), carboplatin (CBDCA) plus nab-PTX, gemcitabine (GEM), S-1, and irradiation (IR). Although Pt. 6, 10, and 15 were well controlled by subsequent chemotherapies (Figure 4C), Pt. 7, 9, 13, and 14 exhibited PD in response to subsequent chemotherapies during follow-up (Figure 4B and Supplemental Figure 2). Prolonged binding of nivolumab on T cells was confirmed for more than 20 weeks after the final dose in all 9 cases (Figure 4 and Supplemental Figure 2). Although the percentage of CB in CD8 T and CD4 T cells decreased in a time-dependent manner, neither the number of nivolumab doses (2–15 doses; Supplemental Table 1) nor the type of subsequent treatment affected the duration of residual binding (Figure 4).

In addition to binding status, we examined two T cell differentiation markers, CD45RA and CCR7, in CD8 and CD4 T cell populations with CB of nivolumab, in order to discriminate 4 subsets: CD45RA^+^CCR7^+^ naive, CD45RA^–^CCR7^+^ central memory, CD45RA^–^CCR7^–^ effector memory, and CD45RA^+^CCR7^–^ effector memory with RA (Figure 4). Several studies demonstrated that the expression pattern of these markers in peripheral blood T cells or tumor-infiltrating T cells correlates with prognosis (15–17) and sensitivity to immune checkpoint inhibitors, including PD-1 blockade (12, 18). Although the number of cases we analyzed in this study was limited, in all patients, the frequency of each subset in the CB population of peripheral blood did not differ significantly for these markers at any time point during long-term follow-up (Figure 4 and Supplemental Figure 2).

To determine the mechanism underlying the long-term residual binding of nivolumab on T cells, we evaluated nivolumab binding on T cells in vitro and also determined the plasma concentration of nivolumab by ELISA (Supplemental Figure 3). Peripheral blood mononuclear cells (PBMCs) obtained from patients before and after a single dose of nivolumab were cultured in nivolumab-free medium, and binding status was evaluated. As a control, we treated PBMCs obtained from patients before nivolumab in vitro and performed a similar experiment (Supplemental Figure 3A). Twenty-four hours after the incubation, nivolumab binding was almost completely lost in CD8 T cells in both the in vitro– and in vivo–treated samples, suggesting that nivolumab binding itself does not last longer than a day (Supplemental Figure 3A). To confirm the presence of residual nivolumab in plasma, we analyzed plasma from 8 representative long-term follow-up patients at the indicated time points, including samples collected after 1 and 2 doses (Supplemental Figure 3B). Although the concentration at later time points was very low in comparison to that after dose 1 and 2, the decrease correlated well with the decrease in the CB ratio (Figure 4 and Supplemental Figure 3B). Importantly, the total percentage of nivolumab-bound T cells varied among patients (Figure 4). The number of nivolumab injections before discontinuation did not correlate with the duration of binding or the plasma concentration (Figure 4 and Supplemental Figure 3B). More importantly, the plasma concentration of nivolumab at the time of complete loss of CB varied among patients (Supplemental Figure 3C). These data suggest that our monitoring technique is more reliable for functional evaluation of the residual efficacy of nivolumab than monitoring of the plasma nivolumab concentration.

### Ki-67 positivity in both total and nivolumab-bound T cells is maintained in patients well controlled by sequential chemotherapy.

**Figure 5:**
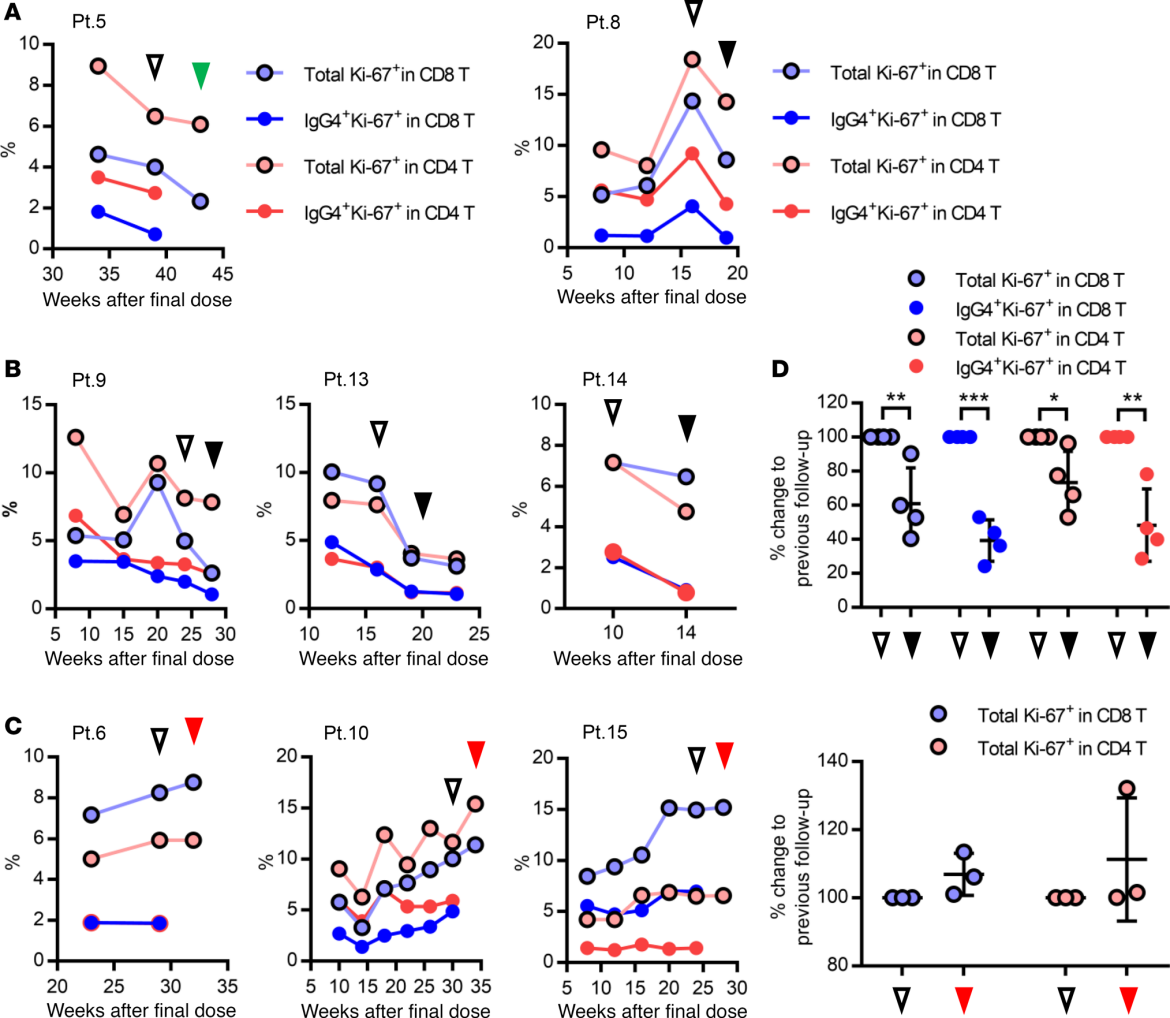
Following up the percentage of Ki-67 positivity in total and nivolumab-bound CD8 and CD4 T cells from patients who underwent sequential treatment. (**A**–**C**) Fresh whole blood samples from 8 non–small cell lung cancer patients were followed up in terms of percentage of Ki-67 positivity in total and nivolumab-bound CD8 and CD4 T cells. Display order is the same as in Figure 4. Black and green triangles indicate the points of progressive disease (PD) and tumor marker re-elevation without an increase in the size of the targeted tumor (as determined by CT scan), respectively. Red triangles indicate the absolute loss of CB of nivolumab in T cells. Unfilled triangles show the follow-up time point previous to those represented by the filled triangles, as described. (**D**) Ki-67 positivity in T cells was compared between 2 time points: at the time of PD (black triangles) versus previous follow-up (unfilled triangles) in Pt. 8, 9, 13, and 14 (top, *n* = 4) and at the time of loss of CB of nivolumab (red triangles) versus previous follow-up (unfilled triangles) in Pt. 6, 10, and 15 (bottom, *n* = 3). Difference was calculated based on the follow-up time point, which was used as a baseline. Data represent mean ± SD. *P < 0.05, **P < 0.01, ***P < 0.001. Total Ki-67^+^ in CD8 T cells, *P* = 0.0013; IgG4^+^ Ki-67^+^ in CD8 T cells, *P* < 0.0001; total Ki-67^+^ in CD4 T cells, *P* = 0.0247; and IgG4^+^ Ki-67^+^ in CD4 T cells, *P* = 0.0029, Student’s *t* test.

Our monitoring strategy allows evaluation of nivolumab-binding status, as well as functional and proliferative markers, in nivolumab-bound T cell populations. To investigate whether immune status in nivolumab-bound T cells after treatment discontinuation can be used to predict either residual efficacy or the synergistic effect with subsequent chemotherapy, we evaluated the percentage of two additional functional markers, Ki-67 and Tbet, which are well-established markers for antitumor immune activity of CD8 and CD4 T cells (13, 14, 19–21), in 8 patients (Figure 4 and Supplemental Figure 4). Because of the limited number of follow-up time points, Pt. 7 was excluded for this analysis (Supplemental Figure 2). Although the percentage of Tbet did not exhibit a specific trend across time points (Supplemental Figure 4), Ki-67 positivity exhibited opposing trends in both total and nivolumab-bound IgG4^+^ T cells in patients whose lung tumors were well controlled by subsequent chemotherapies (Figure 5C) versus patients whose lung tumors progressed (Figure 5, A and B). While all 5 cases (Pt. 5, 8, 9, 13, and 14) exhibited a decrease in Ki-67 positivity in both total and nivolumab-bound IgG4^+^ CD8 T cells at the time of PD or tumor marker re-elevation (Figure 5, A and B), the percentage of Ki-67^+^ T cells in 3 cases (Pt. 6, 10, and 15) whose tumors were well controlled during our follow-up period by single agent nab-PTX or DTX plus RAM, exhibited an increasing or stable trend in Ki-67 positivity until loss of nivolumab CB. We then compared Ki-67 positivity in T cells between two follow-up time points: at the time of PD versus the previous follow-up (Figure 5, A and B), and at the loss of CB versus the previous follow-up (Figure 5C), Ki-67 positivity was significantly lower at the time of PD than at the previous follow-up (*P* = 0.0013, *P* < 0.0001, *P* = 0.0247, and *P* = 0.0029 from left, Student’s *t* test; Figure 5D). These results suggest that Ki-67 positivity in T cells might reflect the residual efficacy of PD-1 blockade, even during the period of subsequent chemotherapy.

### A strategy for monitoring the transcriptome profile in nivolumab-bound CD8 T cells in patients.

**Figure 6:**
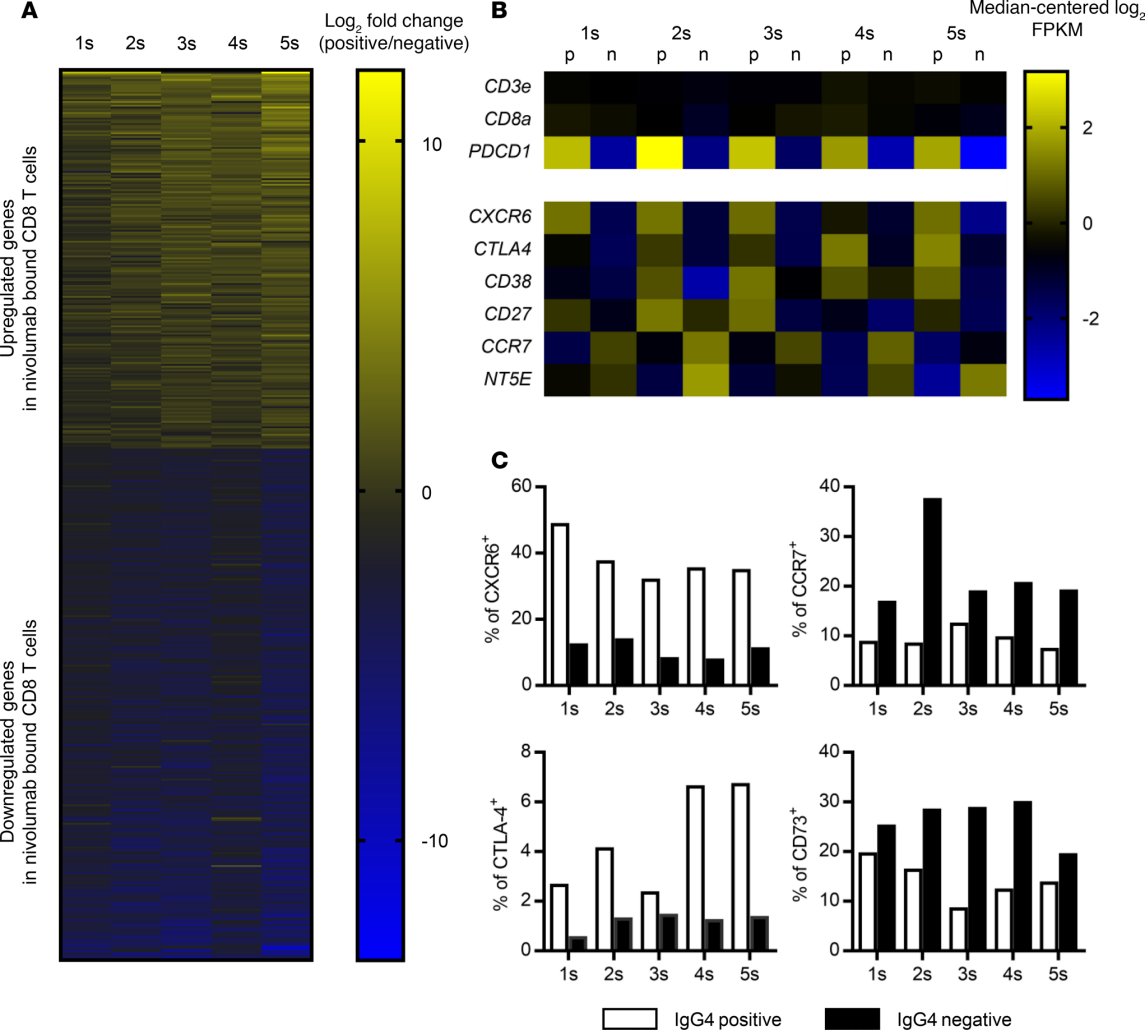
Transcriptome profiling of nivolumab-bound CD8 T cells. (**A**) The transcriptome profiles were compared between nivolumab-bound (IgG4^+^) and nivolumab-unbound (IgG4^–^) CD8 T cells from 5 independent patient samples (Pt. 1s–5s). A total of 485 genes were significantly differentially expressed (Mann–Whitney *U* test, *P* < 0.05). Log_2_ fold change in 5 paired samples of nivolumab-bound/unbound CD8 T cells, shown as a heatmap. (**B**) Representative immune-related genes among the 485 differentially expressed genes. For each gene, expression values across the samples are plotted as log-transformed FPKM values (median-centered log_2_). Nivolumab-bound IgG4^+^ CD8 T cells (p) and nivolumab-unbound CD8 T cells (n) were analyzed. (**C**) Protein levels of CXCR6, CTLA-4, CCR7, and CD73 (encoded by *NT5E*) were evaluated by flow cytometry.

To further characterize the phenotype of nivolumab-bound T cells, we used FACS to isolate IgG4^+^ nivolumab-bound and IgG4^–^ nivolumab-unbound CD8 T cells from 5 different patients 2 weeks after their initial dose (Figure 1 and Supplemental Figure 5A). We generated libraries of entire transcripts and evaluated each of the CD8 T cell populations by RNA sequencing (Supplemental Figure 5B). Based on the transcriptome profile, we identified genes significantly differentially expressed (*P* < 0.05) between the two groups: 206 genes were significantly upregulated and 279 genes were significantly downregulated in the IgG4^+^ nivolumab-bound population relative to the IgG4^–^ nivolumab-unbound population (Figure 6A, Supplemental Table 3, and Supplemental Figure 5B). Representative immune-related genes previously reported to be involved in T cell activation and regulation (15, 22, 23) and cell cycle–related genes are listed in Figure 6B and Supplemental Figure 5C, respectively. To validate our method for extracting the immune profile, particularly in nivolumab-bound CD8 T cells, we confirmed that the expression of *PDCD1* (*PD1*), but not T cell markers *CD3E* and *CD8A*, was clearly enriched in the IgG4^+^ population relative to the IgG4^–^ population (Figure 6B). The gene expression profiles were similar to those of PD-1^hi^ versus PD-1^lo^ CD8 T cells in peripheral blood from a healthy donor (GSE26495) (24), as determined by NextBio analysis (Supplemental Figure 6). Upregulation or downregulation of representative markers (CXCR6, CTLA-4, CCR7, and CD73) was also confirmed at the protein level by flow cytometry (Figure 6C). To investigated whether any of the cell cycle–related gene sets were significantly enriched in the gene expression profiles, we performed Gene Set Enrichment Analysis. Significant enrichment scores (NES) and low *P* values were observed for genes related to “cell division,” “cell cycle G_1_ S phase transition,” and “DNA replication” in nivolumab-bound versus nivolumab-unbound cells (Supplemental Figure 5D). Intrigued by the overlap between the RNA sequencing and flow cytometry data, we performed using Ingenuity pathway analysis to investigate upstream regulators that were activated or inactivated in the nivolumab-bound population in comparison with the unbound population. We identified several different signaling pathways, including prostaglandin E receptor subtype 2 (PTGER2) and VEGF signaling, that were activated in the anti–PD-1 antibody–bound T cells (Supplemental Figure 7).

### Clinical benefit of monitoring nivolumab binding and proliferation in T cells in practical cases.

**Figure 7:**
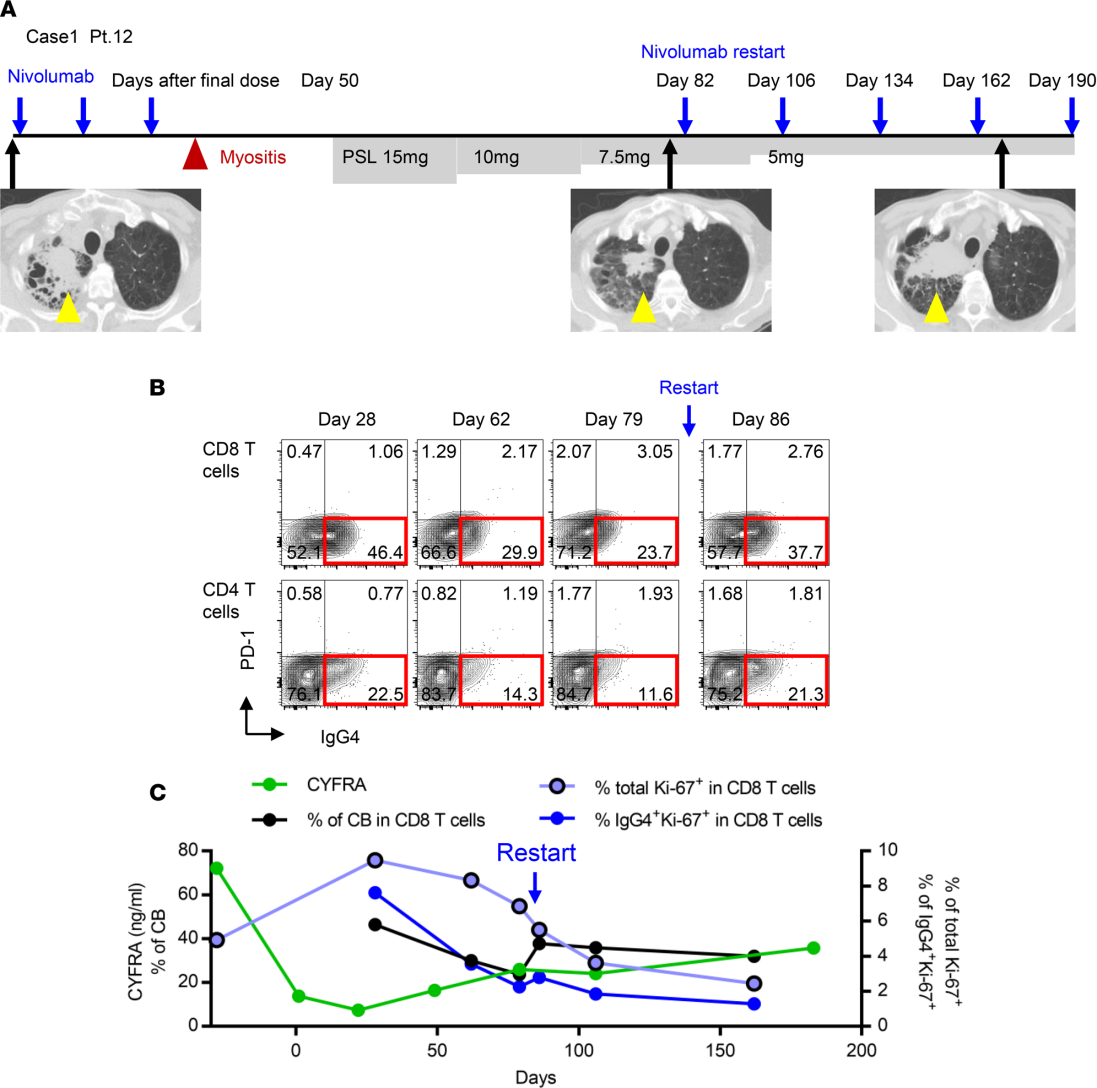
Clinical applications of monitoring of nivolumab binding and Ki-67 positivity in T cells to determine residual efficacy in NSCLC patients. (**A**) Case 1 (Pt. 12): Although the tumor initially responded to nivolumab, treatment was discontinued after 3 doses due to myositis and then restarted after 82 days off. Yellow triangles show tumor in right lung. PSL, prednisolone. (**B**) Nivolumab-binding status in CD8 and CD4 T cells at the indicated time points. (**C**) Tumor marker, CYFRA (ng/ml); percentage of CB of nivolumab in CD8 T cells; and percentage of Ki-67^+^ cells in total and nivolumab-bound CD8 T cells were followed up.

Finally, we present 2 cases in which nivolumab was reinitiated after discontinuation. Both cases showed an initial clinical response to nivolumab, but the clinical course after reinitiation showed the opposite trend, in correlation with T cell proliferation potential (Figure 7 and Supplemental Figure 8).

Case 1 (Pt. 12) exhibited an initial response to second-line PD-1 blockade (yellow arrow, Figure 7A), but nivolumab treatment was discontinued due to an irAE of myositis, and corticosteroid treatment was initiated 50 days after the final nivolumab dose. Because both myalgia and muscle weakness were rapidly ameliorated after initiation of low-dose corticosteroid, and the primary tumor maintained its initial response to nivolumab, we carefully reinitiated nivolumab with steroid tapering 82 days after the initial withdrawal of the drug (Figure 7A). Although the tumors (yellow arrow) did not exhibit an additional response, the symptom of myositis did not recur after reinitiation of nivolumab (Figure 7A). CB of nivolumab in T cells was maintained at all time points, although the percentage of CB increased immediately after reinitiation of nivolumab treatment (Figure 7B). Importantly, Ki-67 positivity in total and IgG4^+^CD8 T cells gradually decreased from day 28 after the final dose, and did not exhibit an increasing trend even after reinitiation of treatment (Figure 7C). A similar trend was confirmed in CD4 T cells as well (data not shown). Given that Ki-67 is a marker for T cell reinvigoration during checkpoint inhibitor treatment (13, 19), our monitoring suggests that the T cells functioning in both antitumor immunity and autoimmunity could not be re-activated in this patient once they had been strongly suppressed by corticosteroids. Subsequently, chemotherapy was initiated as a third-line treatment.

Case 2 (Pt. 5) initially responded well to nivolumab treatment (yellow arrow, Supplemental Figure 8A); however, treatment was discontinued 22 weeks after the initial dose due to an irAE of thrombocytopenia with elevated titer of platelet-associated antibodies (PA-IgG) (Supplemental Figure 8B). After nivolumab discontinuation, platelet counts stabilized and clinical response was maintained for 40 weeks, and CB of nivolumab was sustained until 38 weeks (Figure 3C). At 43 weeks after the final dose, we confirmed the absolute loss of CB of nivolumab in T cells (Figure 3C) and a decrease in Ki-67 positivity in T cells (Figure 5A). Around this time point, the serum carcinoembryonic antigen (CEA) concentration increased again, whereas both platelet count and PA-IgG titer returned to their normal ranges (Supplemental Figure 8, B and C). We concluded that the residual binding of nivolumab had completely disappeared by this point and restarted nivolumab treatment with careful monitoring of thrombocytopenia. This patient exhibited a durable response to the restart of nivolumab (Supplemental Figure 8A), although the tumor did not clearly shrink, as it did in response to the first course of nivolumab. In terms of reactivation of antitumor response, elevation of CEA temporary stopped, and the level of CYFRA was maintained within the normal range (Supplemental Figure 8B). After the restart of nivolumab, Ki-67 re-elevated at 2 time points, 2 and 6 weeks after reinitiation of nivolumab (Supplemental Figure 8C). In terms of reactivation of autoimmunity, we saw a 1-time re-elevation of PA-IgG titer 24 weeks after nivolumab reinitiation, but thrombocytopenia was not clearly correlated with PA-IgG titer at that time point. Because the tumor exhibited regrowth with elevation of both CEA and CYFRA and symptoms of shoulder pain, nivolumab was discontinued 85 weeks after the final injection of the first course of nivolumab.

## Discussion

Our simplified method was feasible in a real-world cohort and substantiated both the long-term duration of nivolumab binding beyond 20 weeks and changes in binding status in fresh whole blood after drug discontinuation. Although long-term extension of nivolumab in plasma was considered sufficient to maintain the binding on T cells, plasma concentration can be affected by several factors. These include pharmacokinetics in each patient, total PD-1 positivity on T cells throughout the body, and immune cell activation status to process nivolumab. This study also demonstrated that the residual binding of nivolumab itself does not necessarily indicate a T cell–reinvigorated status and that monitoring with functional markers, including Ki-67, is essential for determining the actual residual efficacy of nivolumab. Therefore, our combinational strategy, monitoring nivolumab binding and proliferation status of T cells, is more reliable for determining residual efficacy in regard to both activated antitumor immunity and autoimmunity than monitoring the plasma concentration of nivolumab alone.

Recent clinical studies have shown that the combination of anti–PD-1 antibody and anti–CTLA-4 antibody or platinum doublet chemotherapy results in better outcomes than anti–PD-1 monotherapy (25–27), suggesting that there is room to improve the efficacy of anti–PD-1 monotherapy with appropriate combination therapies (28). However, it remains unknown which therapeutic drugs and regimens are the most suitable synergistic partners. Schedules for these combination treatments also need to be optimized based on the clinical characteristics of individual patients, including tumor microenvironment and tolerability. Our results showed that the sequential chemotherapeutic regimens did not affect the prolonged binding of nivolumab after discontinuation and that T cell proliferation potential could be monitored during chemotherapies, at least in the small number of cases studied. Therefore, monitoring of nivolumab binding by staining for Ki-67 and other functional markers in T cells might aid in evaluating the prolonged efficacy of PD-1 blockade after discontinuation and might also help monitor the benefit of PD-1 blockade in concurrent treatment with chemotherapies, which will become the first-line treatment in the near future (26, 29, 30). Different chemotherapies can influence host antitumor immune responses in different ways. For instance, some reagents can induce immunogenic cell death in the tumor microenvironment, leading to innate immune cell activation by releasing damage-associated molecular patterns and increased antigen presentation to T cells (31, 32). Other treatments can abrogate immune-suppressive cells, such as regulatory T cells and myeloid cells, thereby improving the cytotoxic function of CD8 T cells (33, 34). In future studies, our monitoring method could help systematically determine which chemotherapeutic regimens should be used after, or in combination with, immune checkpoint treatment.

Our transcriptome analysis revealed that the PTGER2 and VEGF signaling pathways, in addition to several signaling pathways that are known for upregulating PD-1 expression, such as FoxO1, NF-κB, and NFAT, were enhanced in the antibody-bound IgG4^+^ population in comparison with the IgG4^–^ population (35). Recent studies showed that VEGF regulates PD-1 expression (36) and PGE2 coordinately impairs cytotoxic T cell function with PD-1 signal (37) and that inhibition of both pathways improves the efficacy of immune checkpoint inhibitors (38, 39). These observations suggest that our approach is highly efficient for detecting active pathways in the T cell populations responsible for antitumor immune response or irAEs as well as for determining potential combination-therapeutic targets to synergize with PD-1–blocking therapy or prevent adaptive resistance (40). Future studies will focus on comparing the T cell receptor sequence, particularly in nivolumab-bound T cells, longitudinally in the same patients who develop dramatic antitumor response, hyperprogressive disease (41), or severe irAEs, thereby identifying the specific characteristics of T cells that are responsible for these clinical phenotypes.

To evaluate the potential for extended application of our method in actual clinical settings, we described two clinical cases in which nivolumab was reinitiated. In the first patient, who did not exhibit a clinical response after reinitiation of nivolumab, Ki-67 positivity closely reflected the reinvigoration status of T cells, which were not reactivated, even after reinitiation of anti–PD-1 blockade, suggesting that our strategy could be used to monitor the residual efficacy of nivolumab during corticosteroid treatment. In the second patient, in whom both tumor marker re-elevation and recovery from thrombocytopenia emerged at the time of absolute loss of CB of nivolumab, nivolumab-binding status closely reflected the residual efficacy of antitumor immunity and autoimmunity. Although the second case did not exhibit clear tumor shrinkage in response to reinitiation of nivolumab (Supplemental Figure 8), we were able to treat the patient for almost 6 months without re-elevation of tumor markers and regrowth of tumor by images. In comparison to the reinitiation of the antitumor effect, there was no clear autoimmune effect, i.e., the reincrease of PA-IgG titer and redecrease of platelet count did not occur in parallel. This phenotype has also been reported by another group (42). In contrast to the first patient, who did not exhibit an increase in Ki-67 in T cells in response to reinitiated nivolumab, the second patient exhibited both induction of Ki-67^+^ T cells and a durable response to reinitiated nivolumab. A finding in just 2 cases is not sufficient to conclude that corticosteroid diminishes the antitumor effect, because a certain number of patients maintain the efficacy of anti–PD-1 blockade, even after discontinuation of this treatment under corticosteroid therapy. Therefore, we will continue to investigate how corticosteroid modulates the antitumor response by PD-1 blockade in each clinical setting and determine whether it is possible to dissect antitumor immune response versus autoimmune response using our method along with sequencing of T cells bound by anti–PD-1 antibody.

A similar strategy for detecting anti–PD-1 antibody binding was very recently published by another group (11, 13). By contrast, our study focused on monitoring the binding and activation status of nivolumab-bound T cells using fresh whole blood from NSCLC patients after discontinuation of treatment.

Targeted therapy or chemotherapy drugs have short half-lives in patients. In comparison with these agents, antibodies, especially PD-1–blocking antibodies, have longer half-lives and efficacy, even several months after the last dose. The antibody concentration in serum or plasma does not reflect the functional efficacy of this drug as reliably as PD-1–blocking antibody on T cells. Our monitoring method is very simple, requiring only a small amount of fresh blood, making it readily applicable to the clinical setting. Because patients with several types of malignancies will undergo other treatments after, or concurrent with, anti–PD-1–blocking treatment, monitoring of T cell proliferative status along with therapeutic antibody binding could help evaluate the efficacy of PD-1 blockade in a variety of clinical settings. Given the limitations of this approach in terms of the number of parameters analyzed, future studies should focus on integrating newer technology into our analysis.

## Methods

### Patient sample collection.

Sampling was performed during routine clinical procedures. All research on human subjects was performed in accordance with the above protocols. Patient eligibility criteria are shown in the Supplemental Methods. This study was registered in the University Hospital Medical Information Network Clinical Trials Registry (UMIN000024623).

### Study design and sample analysis protocol.

Sixty NSCLC patients who began nivolumab treatment as the second-line or further-line treatment at Osaka University Hospital and Toneyama Hospital between January 2016 and May 2017 were enrolled in this study (Supplemental Table 1). Forty-two and eighteen patients, respectively, started nivolumab from January 2016 to October 2016 (period 1) and November 2016 to May 2017 (period 2). Twenty-seven cases were treated with second-line treatment, and 33 cases with third- or further-line treatment. Five cases in period 2 were selected for T cell sorting and transcriptome profiling through RNA sequencing. Long-term follow-up was possible in 9 of the patients enrolled (Supplemental Table 1). These patients were followed up every 4 ± 1 weeks at times more than 8 weeks after the final injection of nivolumab, until the loss of CB of the antibodies (Supplemental Table 1). Clinical characteristics of all patients analyzed in this study are shown in Supplemental Table 2.

### Immune analysis of patient samples.

Freshly collected blood samples were treated with Fc block (Miltenyi Biotech) and then subjected to red blood cell lysis (Thermo Fisher Scientific, eBioscience RBC Lysis Buffer). After spinning and washing with 2% FBS in PBS, cells were subjected to surface staining. For freshly collected effusion samples, the cells were subjected to red blood cell lysis after spinning, passed through a 40-μm cell strainer (FALCON), and stained. To detect nivolumab binding, samples were incubated with anti–human IgG4-PE or isotype control (Abcam) after Fc blocking. Antibody clone numbers for immune analysis are listed in the Supplemental Methods. Fixation/permeabilization buffers (Thermo Fisher Scientific, eBioscience Foxp3/Transcription Factor Staining Buffer Set) were used for intracellular staining.

### Cell line studies.

HEK293T cells were purchased from ATCC. HEK293T cells were grown in DMEM (Nacalai Tesque) supplemented with 10% FBS, penicillin (100 U/ml), and streptomycin (100 mg/ml). Expression plasmid for human PD-1 (catalog HG10377-UT, Sino Biological) was transfected into HEK293T cells using Lipofectamine 2000 (Life Technologies). Expression of PD-1 and nivolumab binding through human IgG4 were evaluated by flow cytometry. Isotype controls for PD-1 and human IgG4 were purchased from BD Biosciences and Abcam, respectively (Supplemental Methods). Nivolumab and human IgG4 isotype control were purchased from Bristol-Myers Squibb/Ono and Biolegend, respectively. The binding capacity of recombinant human PD-L1 to PD-1–expressed HEK293T was evaluated after incubation with serial dilutions of nivolumab. Recombinant human PD-L1–Fc and secondary anti–human IgG1-FITC antibody were purchased from R&D Systems and Abcam, respectively. Acquisition of samples was performed on a BD Canto II cytometer equipped with Diva software, and data were analyzed using FlowJo.

### ELISA for plasma concentration of nivolumab.

Ninety-six-well plates (NUNC) were coated with a recombinant human PD-1–Fc (R&D Systems) at 0.2 μg/ml in PBS and incubated overnight at 4°C. Then, the plates were blocked with 5% skim milk (BD Biosciences) in PBS containing 0.05% Tween-20 (blocking buffer) for 1 hour. Plasma samples from patients were pretreated with blocking buffer overnight before ELISA experiments. Next, plasma samples diluted 1:100 and 1:2,000 in blocking buffer were applied to each well and incubated for 2 hours. After washing, the plates were incubated for 1 hour in HRP-conjugated secondary antibody for human IgG4 (Southern Biotech) diluted 1:2,000 in blocking buffer and then stained with TMB Substrate Reagent (BD Biosciences). The reaction was stopped with 1 M H_2_SO_4_, and absorbance was measured. Because human plasma samples exhibited a background reaction, serial dilutions of nivolumab in PBS (1:5 dilutions, starting from 0.5 μg/ml) were as a standard on each plate to calculate the concentration relative to pure nivolumab.

### T cell sorting and RNA sequencing.

Mononuclear cells were enriched using Ficoll-Paque (GE Healthcare). Sorting of nivolumab-bound peripheral blood T cells (CD45^+^CD56^–^CD19^–^TCRab^+^CD8a^+^IgG4^+^) was performed on a BD FACSAria II cell sorter; the gating method is shown in Supplemental Figure 5A. RNA was prepared from sorted CD8 T cells (2 × 10^4^ to 5 × 10^4^ cells/each population) using the Qiazol and miRNeasy Mini kits (Qiagen). Library preparation was performed using the TruSeq stranded mRNA sample prep kit (Illumina). For small-scale samples, cDNA was generated using the Clontech SMART-Seq v4 Ultra Low Input RNA Kit (Takara Clontech). Each cDNA sample was sheared (200–500 bp) on a Covaris S220 (Covaris) and prepared using the KAPA Library Preparation Kits (Kapa Biosystems) for 75-bp single-end reads. Sequencing was performed on an Illumina HiSeq 2500 platform in a 75-base single-end mode. RNA sequence data analysis was performed as described in the Supplemental Methods.

### Statistics.

Numerical data are presented as mean ± SD. For comparisons of two groups, data were analyzed using 2-tailed unpaired Student’s *t* test. A *P* value of less than 0.05 was considered significant.

### Study approval.

All patient samples were obtained from subjects who provided informed consent for blood and effusion collection in accordance with the Declaration of Helsinki and with approval from the ethical review board of the Graduate School of Medicine, Osaka University (no. 15383) and Toneyama National Hospital (no. 1545).

## Author contributions

AO, TU, SK, PSH, KF, MM, TK, and AK conceived and designed the experiments. AO, TU, SK, KF, TT, HH, YY, SY, YK, YN, TO, M. Kanazu, M. Kuroyama, MH, TK, YF, MI, YS, YA, KI, IN, YT, and HK performed collection of patient samples and experiments. AO, SK, DO, YN, and HM collected and analyzed data. AO, SK, TK, and AK wrote the manuscript. HT, EAA, PSH, KKW, and GD provided technical support and provided reagents and conceptual advice. All authors contributed to the final manuscript.

## Supplementary Material

Supplemental data

## Version 1 10/04/2018

Electronic publication
