# Supplementary material for: Clinical implications of monitoring nivolumab immunokinetics in non–small cell lung cancer patients
Source: JCI Insight. 2018 Oct 4;3(19):e59125. doi: 10.1172/jci.insight.59125 (PMC6237460; doi:10.1172/jci.insight.59125)
Supplement: Supplemental data [file jciinsight-3-59125-s103.pdf]

Supplementary Information

Clinical implications of monitoring nivolumab immunokinetics in previously treated non–small cell lung cancer patients

Akio Osa, Takeshi Uenami, Shohei Koyama, Kosuke Fujimoto, Daisuke Okuzaki, Takayuki Takimoto, Haruhiko Hirata, Yukihiro Yano, Soichiro Yokota, Yuhei Kinehara, Yujiro Naito, Tomoyuki Otsuka, Masaki Kanazu, Muneyoshi Kuroyama, Masanari Hamaguchi, Taro Koba, Yu Futami, Mikako Ishijima, Yasuhiko Suga, Yuki Akazawa, Hirotomo Machiyama, Kota Iwahori, Hyota Takamatsu, Izumi Nagatomo, Yoshito Takeda, Hiroshi Kida, Esra A. Akbay, Peter S. Hammerman, Kwok-kin Wong, Glenn Dranoff, Masahide Mori, Takashi Kijima, Atsushi Kumanogoh

Supplemental Figures 1 – 8

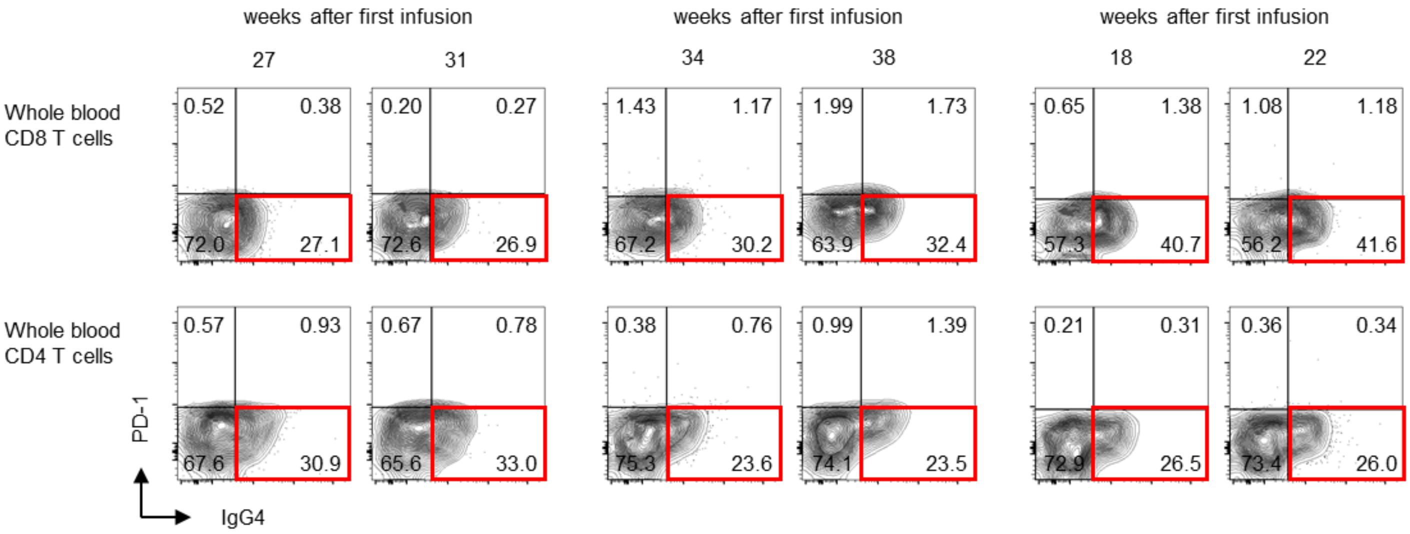

Supplemental Figure 1. The frequency of nivolumab-bound T cells was maintained in patients who continued treatment.

Nivolumab binding in CD8 and CD4 T cells was analyzed at two follow-up points, as indicated, in fresh peripheral blood from three representative cases from protocol 1 that continued treatment.

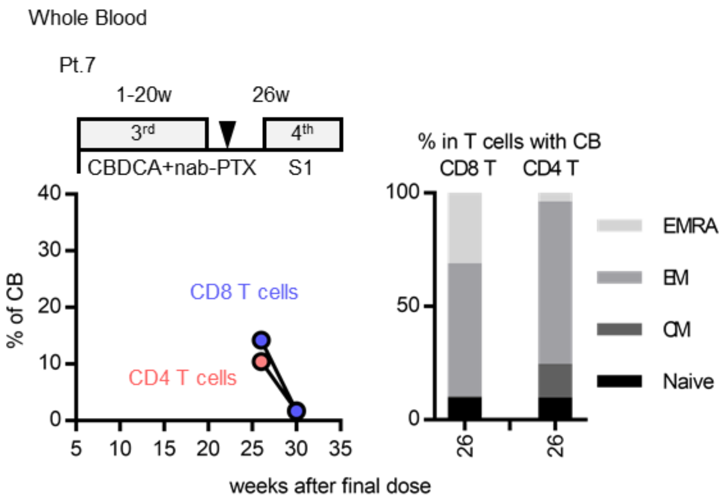

Supplemental Figure 2. Long-term follow-up of nivolumab binding to T cells from fresh whole blood.

Nivolumab binding was followed up in fresh peripheral blood from an additional case, Pt.7.

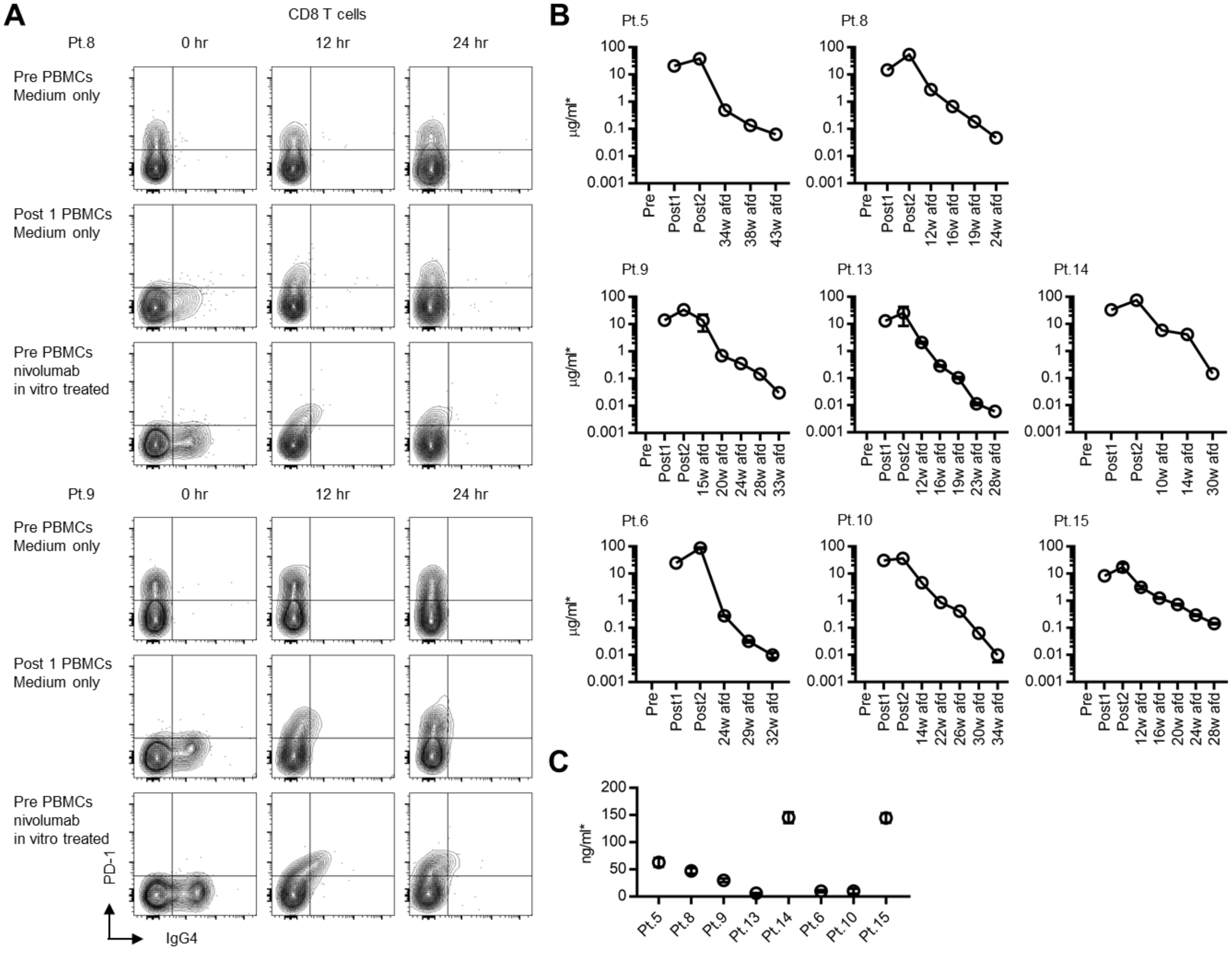

Supplemental Figure 3. Long-term duration of nivolumab binding is due to sustained circulation of residual nivolumab in plasma.

(**A**) PBMCs acquired from Pt.8 and 9 at pretreatment (pre PBMCs) and after a single dose (post 1 PBMCs) were cultured in regular medium without nivolumab (top and middle). Pre PBMCs were also incubated with 10 μg/ml nivolumab in vitro before the cultures were started (bottom). Nivolumab binding status was monitored at the indicated time points. (**B**) Concentrations of nivolumab in plasma from patients at the indicated time points when binding on T cells was analyzed (afd: after final dose). Data indicate three replicate wells and are representative of three independent experiments. (**C**) Plasma level of nivolumab at the time of absolute loss of CB (last follow-up point). *Units are calculated based on the concentration of nivolumab in PBS, as described in Methods (**B,C**).

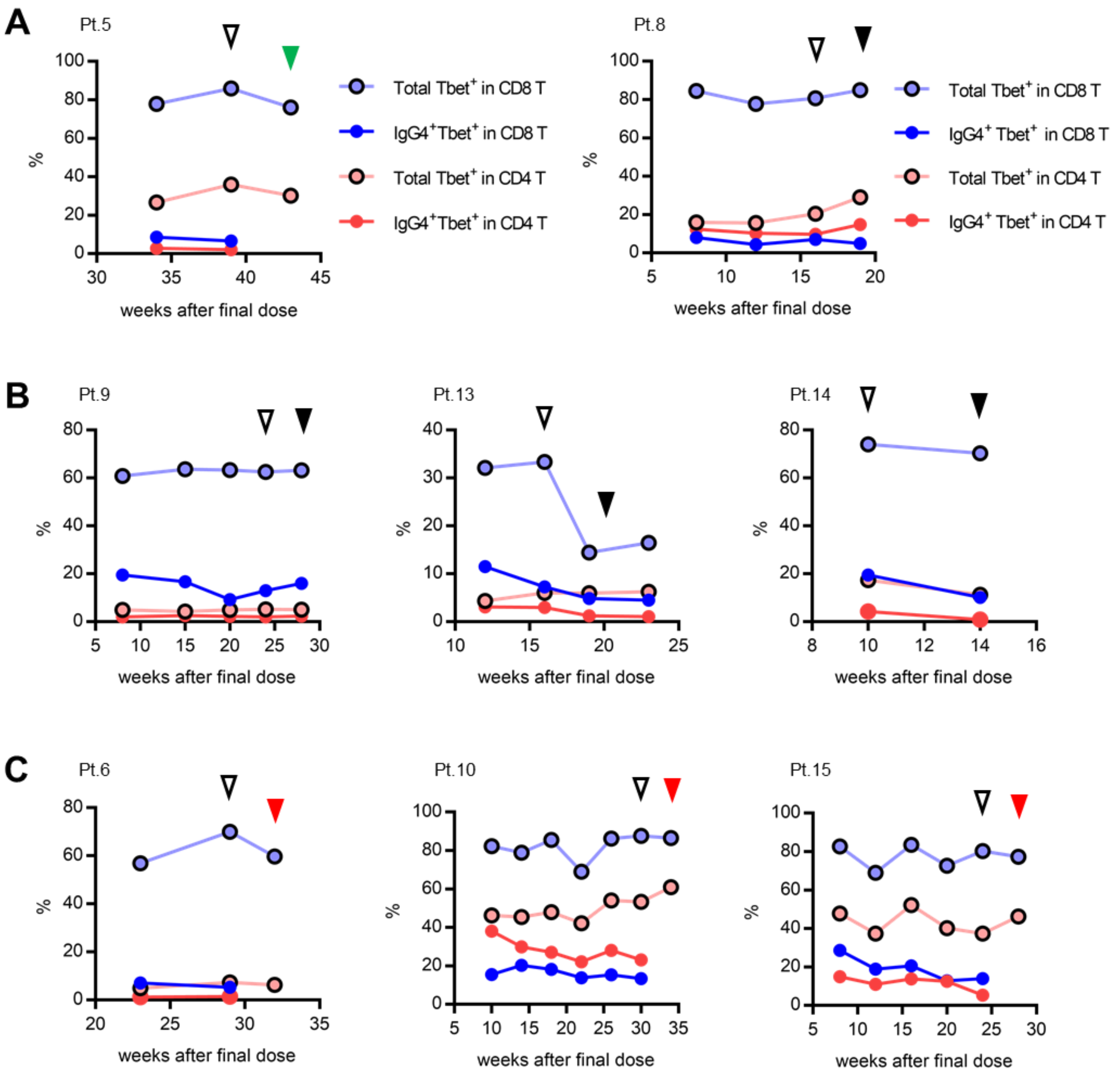

Supplemental Figure 4. Tbet positivity in total and nivolumab-bound CD8 and CD4 T cells in patients who underwent sequential treatment.

A, B, C. Tbet positivity in total and nivolumab-bound CD8 and CD4 T cells was followed up in fresh whole blood samples from eight patients with non–small cell lung cancer. Display order of patients is the same as in Figure 4. Black and green arrows show points of progressive disease and tumor marker re-elevation without increase in the size of the targeted tumor (as determined by CT scan), respectively.

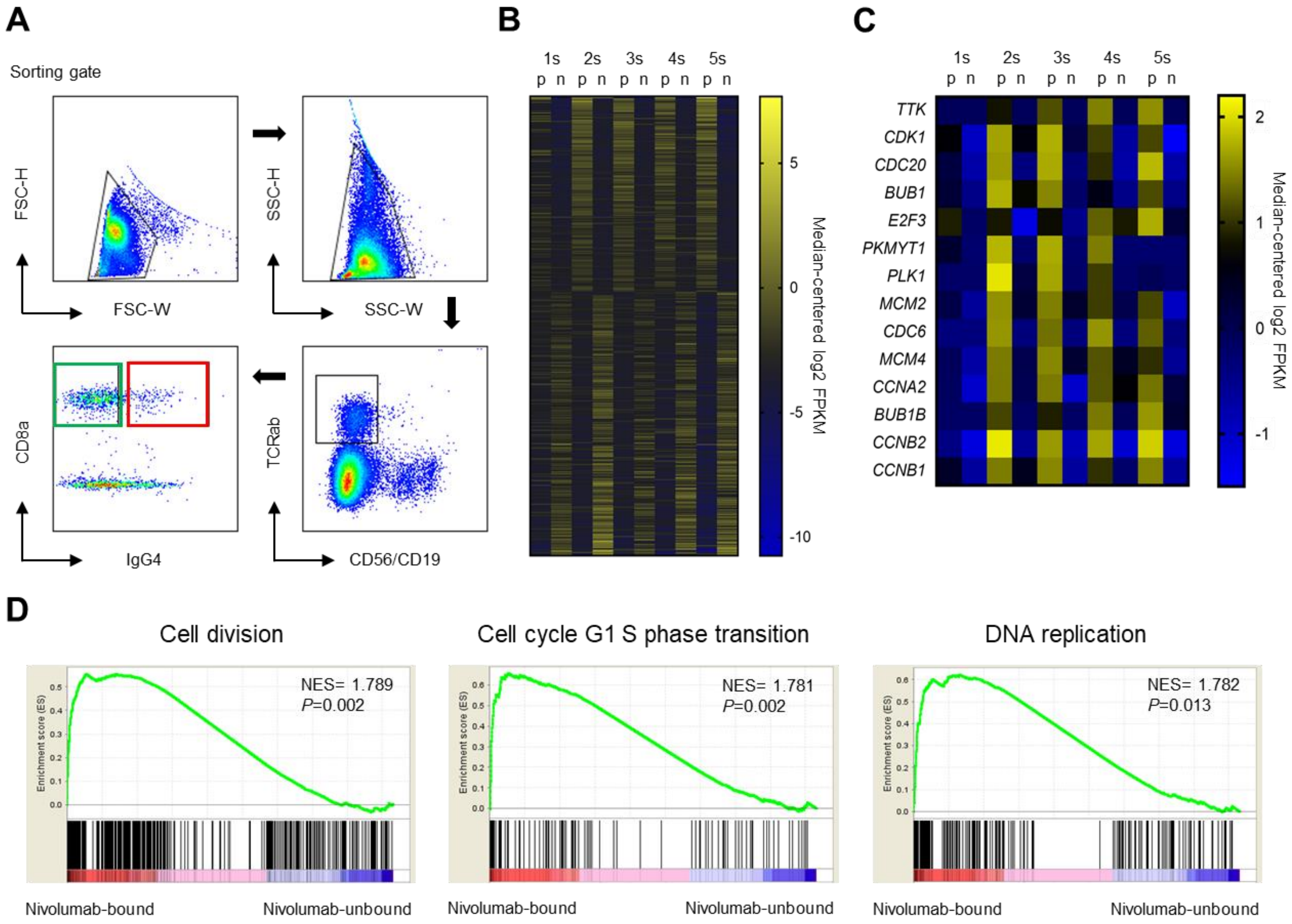

Supplemental Figure 5. Sorted nivolumab-bound CD8 T cells used for transcriptome profiling.

(**A**) Gating strategy for sorting $CD45^{+}CD56^{-}CD19^{-}TCRab^{+}CD8a^{+}IgG4^{+\text{ or }-}$ T cells. (**B**) A total of 206 genes were significantly upregulated, and 279 were significantly downregulated, in nivolumab-bound CD8 T cells ($IgG4^{+}$) vs. nivolumab-unbound cells ($IgG4^{-}$). Statistical significance was determined by Mann–Whitney U test ($p<0.05$). (**C**) Fourteen representative cell cycle–related genes were upregulated in nivolumab-bound cells. Cell cycle–related genes were identified based on the “cell cycle process” Gene Ontology annotation in MSigDB. (**D**) Enrichment plots from GSEA conducted on RNA-Seq expression datasets. Significant enrichment scores (NES) and low p-values were observed for genes related to “Cell division,” "Cell cycle G1 S phase transition,” and "DNA replication" in nivolumab-bound vs. nivolumab-unbound cells.

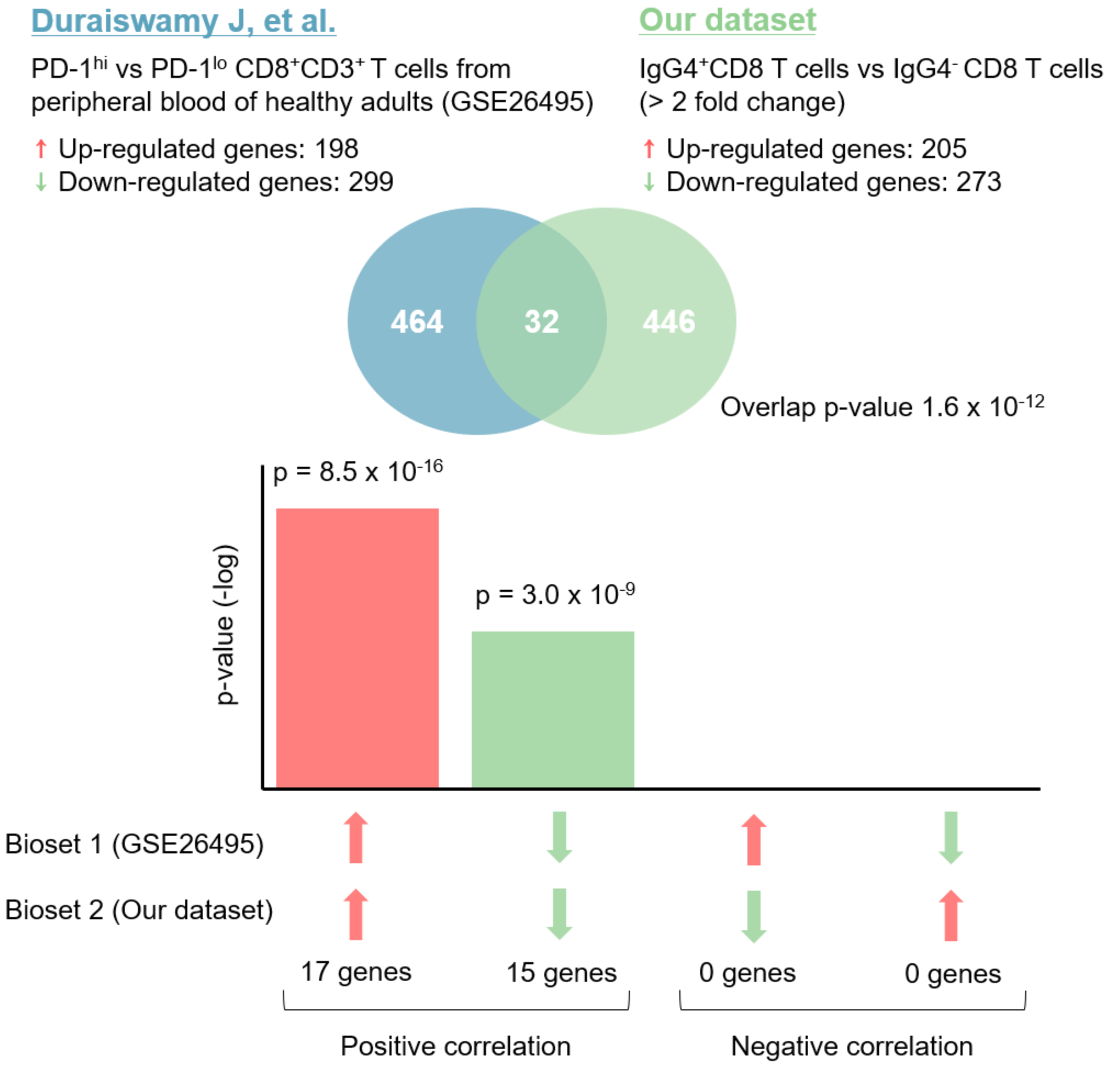

Supplemental Figure 6. NextBio analysis of RNA-Seq data from nivolumab-bound vs. unbound CD8 T cells.

Using NextBio analysis, genes differentially expressed in nivolumab-bound ($IgG4^+$) and unbound ($IgG4^-$) CD8 T cells were compared with those in PD-1^{hi} and PD-1^{lo} CD8 T cells from healthy donors. Duraiswamy J, et al. (2011): data obtained from a public repository (GSE26495). Our dataset included RNA-seq data from this study, in which 478 of the 485 genes up- or down-regulated > 2.0-fold in IgG4-negative vs. IgG4-positive CD8 T cells were recognized and could be interpreted by NextBio. Venn diagram shows the number of common and unique genes in both sets. Bar graphs show overlap p-values for genes up-regulated (red arrows) or down-regulated (blue arrows) under each condition. The y-axis scale represents −log (p-value).

Upstream regulator	Predicted activation state	Activation z-score	p-value of overlap	Target molecules in dataset
PTGER2	Activated	5.19	1.67E-23	*ADGRE1,ASPM,BUB1B,CCNA2,CCNB2,CCR7,CDH1,CDKN3,CENPF,CEP55,CKAP2L,DEPDC1,ECT2,IL10,KIF11,KIF15,KIF20A,KIF2C,MELK,MKI67,NEK2,NUF2,NUSAP1,PBK,PLK1,TPX2,TROAP,TTK*
TBX2	Activated	4.24	1.42E-14	*ANLN,ASF1B,AURKB,BUB1,CCNA2,CCNB1,CDC6,CDCA3,CDCA5,CDH1,CDK1,CDT1,E2F7,FOXM1,MCM2,MCM4,PKMYT1,PLK1*
FOXM1	Activated	4.17	3.37E-18	*AURKB,AXIN2,BIRC5,BUB1B,CCNA2,CCNB1,CCNB2,CDC20,CDC25A,CDCA2,CDH1,CDK1,CDKN3,CENPF,ESR1,FOXM1,KIF20A,LEF1,MKI67,MYC,NEK2,PLK1,PLK4,STMN1,TOP2A*
AREG	Activated	3.99	3.18E-11	*AURKB,BIRC5,CCNA2,CCNB1,CCNB2,CDC20,CENPF,FOXM1,HJURP,KIF14,KIF20A,MKI67,MYBL2,PLK1,RRM2,TOP2A*
CSF2	Activated	3.88	1.28E-20	*ANLN,BIRC5,BUB1,BUB1B,CCL4,CCNA2,CCNB1,CCR5,CCR7,CD38,CD74,CDC20,CDCA2,CDCA3,CDCA5,CDH1,CDK1,CISH,CTLA4,DSCC1,E2F7,EXO1,FCGR2B,FOXM1,HJURP,IL10,IL2RA,IL5RA,KIF11,MKI67,MYC,NEK2,NUSAP1,OSM,PLK1,PTGER2,RRM2,SKA1,SOCS2,STMN1,TLR5,TNFRSF11A,TNFRSF4,TNFRSF9,TOP2A,TPX2,UBE2C,UHRF1*
HGF	Activated	3.88	7.89E-13	*AKAP12,AURKB,BIRC5,BUB1,BUB1B,CCL4,CDC20,CDC25A,CDC6,CDH1,CDK1,CDKN3,CENPF,DUSP4,EDA,FOXM1,HMMR,HMOX1,IL10,IL5RA,KIF11,KIF15,KIF2C,MCM2,MELK,MKI67,MYC,NDC80,NEK2,PKMYT1,PLK1,PLK4,RUNX2,SOCS2,SPRY2,TPX2,TTK,UBE2C,VCAM1*
RABL6	Activated	3.80	1.21E-25	*AURKB,BUB1,BUB1B,CCNA2,CCNB1,CDC25A,CENPF,HMMR,HMOX1,KIF23,MCM10,MCM2,MELK,MYC,NCAPG,NDC80,NEK2,PBK,PLK1,PMAIP1,POLE2,TOP2A,TPX2,TTK,UBE2C*
EP400	Activated	3.72	1.08E-15	*CCNA2,CDC20,CDC25A,CDC6,CDCA3,CENPF,E2F7,FOXM1,MAPK12,MCM4,MYBL2,NEK2,PLK1,SKA1,UHRF1*
MITF	Activated	3.70	5.02E-09	*AURKB,CCNB1,CDCA3,CDT1,CENPF,CEP55,ECT2,ESPL1,KIF20A,KIF4A,KIFC1,LYST,MCM2,MCM4,NGFR,NUF2,PLK1,POLE2,SPC24,STXBP1,TPX2,UBE2C*
MYC	Activated	3.61	8.55E-05	*ABCB9,ACTN1,ACVR2A,AKAP12,AURKB,BCAT1,BIRC5,BUB1,BUB1B,CCNA2,CCNB1,CCNB2,CDC20,CDC25A,CDH1,CDK1,DUSP4,E2F3,ERBB2,FABP5,FOXM1,GLS2,HMOX1,IL10,IL5RA,JAG2,LIMS1,MKI67,MYC,PLK1,PMAIP1,RARG,RRM2,SCAMP5,SEMA4A,SMS,STMN1,TF,TLE1,TYMS,UBE2C,VCAM1*
E2f	Activated	3.39	7.78E-10	*CCNA2,CCNB1,CDC25A,CDC6,CDK1,GINS2,MCM10,MCM2,MCM4,MYBL2,MYC,NUSAP1,RRM2,TPX2,TTK,TYMS,UBE2C*
ERBB2	Activated	3.25	3.96E-18	*ASPM,BIRC5,BUB1,BUB1B,CAVIN3,CCL4,CCNA2,CCNB1,CCNB2,CDC20,CDC25A,CDC6,CDCA2,CDCA3,CDCA5,CDH1,CDK1,CDKN3,CDT1,CENPF,CHD7,CLEC11A,COL6A1,CYP2J2,DLL1,E2F3,E2F7,EPHX2,ERBB2,ESPL1,ESR1,ETV1,FZD4,GINS1,GINS2,JAG2,MCM10,MCM2,MCM4,MKI67,MYBL2,MYC,NCAPG,NDC80,NEK2,PFN2,POLE2,POLQ,RAD51AP1,RARG,RRM2,SOCS2,SOWAHC,SPINT2,TOP2A,TYMS,UBE2C,ZWINT*
CD40	Activated	3.24	7.71E-07	*BIRC5,CCL4,CCNA2,CD27,CD38,CD70,CR2,CTLA4,FCGR2B,IL10,IL2RA,IL5RA,MYC,PDCD1,PTGER2,RGS13,TNFRSF9,VCAM1*
E2F3	Activated	3.21	8.34E-10	*BAIAP2L1,BIRC5,CCNA2,CCNB1,CCNB2,CDC25A,CDC6,CDCA3,CDK1,E2F3,MCM10,MCM2,MCM4,MYBL2,MYC,PCLAF,PLK1,RRM2,TPX2,UBE2C*
CCND1	Activated	3.02	1.34E-17	*ASPM,BIRC5,CCNA2,CDC6,CDCA2,CDH1,CENPF,CEP55,CLSPN,DDIAS,DEPDC1,DTL,E2F7,EPGN,EPS8,ERBB2,FAM83D,FOXM1,HJURP,KIF11,KIF20A,KIF2C,KIF4A,KNL1,KRT1,MCM10,MCM4,MELK,MYC,NT5E,PCLAF,PLK1,RRM2,SATB1,STXBP1,TPX2,TYMS,UHRF1*
ELAVL1	Activated	2.97	4.65E-05	*CDC6,HMOX1,KIF11,MYBL2,MYC,NCAPG,NEK2,NUF2,PBK,TOP2A*
FOXO1	Activated	2.94	6.86E-14	*AFF3,ANLN,ASPM,ATP6V0A1,BACH2,BIRC5,CCL4,CCNA2,CCNB1,CCNB2,CCR7,CD70,CDK1,CENPF,DEPDC1,DLGAP5,EFHD1,FOXM1,GZMA,HMOX1,IRS2,KIF11,KLF7,MYC,NCAPG,NEK2,NUSAP1,PBK,PMAIP1,RUNX2,SCD,SPRY2,TNFRSF11A,TNFRSF9,TNFSF9,VCAM1*
TICAM1	Activated	2.75	0.00878	*CCL4,CD38,CXCL13,IL10,MCM2,MKI67,TLR3,VCAM1*
RARA	Activated	2.69	3.58E-05	*AASS,ASPM,BIRC5,CCNA2,CD38,CDK1,CDKN3,CENPF,CXCR5,E2F7,IL5RA,KIF23,KNL1,MND1,MYC,NCAPG,PBK,PLK4,RARG*
Vegf	Activated	2.66	3.26E-15	*AKAP12,AURKB,AXIN2,BIRC5,BUB1,BUB1B,CDC20,CDC25A,CDC6,CDH1,CDK1,CDKN3,CENPF,CSGALNACT1,DUSP4,EDA,FOXM1,HMMR,HMOX1,IL5RA,KIF11,KIF15,KIF2C,LEF1,MCM2,MELK,MKI67,MYC,NDC80,NEK2,P2RY14,PKMYT1,PLK1,PLK4,PMAIP1,SOCS2,TLR3,TNFRSF11A,TNFRSF9,TPX2,TTK,UBE2C,VCAM1*
SMOC2	Activated	2.63	1.76E-10	*AURKB,CCNB1,CDH1,CDK1,CENPF,MCM10,MCM4*
YAP1	Activated	2.62	1.67E-07	*ANLN,AURKB,BIRC5,CDH1,CDK1,FOXM1,MYC,NEK2,PDGFB,PLK1,PMAIP1,UHRF1,VCAM1*
MTOR	Activated	2.59	0.0157	*BIRC5,CCR7,CDH1,FABP5,IL10,IRS2,MYC,PRX,RUNX2,TLR5,UBE2C,VCAM1*
VEGFA	Activated	2.55	0.00516	*BIRC5,CTLA4,DLL1,DUSP4,E2F3,HMOX1,MKI67,PDCD1,PDGFB,RUNX2,TNFRSF9,UNC5B,VCAM1*

S100A6	Activated	2.45	1.63E-06	CDH1,CDK1,CDKN3,DEPDC1,NEK2,PBK,TYMS
E2F2	Activated	2.45	2.14E-10	BIRC5,CCNA2,CCNB1,CCNB2,CDC25A,CDC6,CDK1,E2F3,MCM10,MCM2,MCM4,MYBL2,MYC,RRM2
E2F1	Activated	2.42	9.45E-10	AURKB,BIRC5,CCNA2,CCNB1,CCNB2,CDC20,CDC25A,CDC6,CDK1,DUSP4,E2F3,ECT2,FOXM1,GINS1,IRS2,JAG2,KIF23,KRT1,MCM10,MCM2,MCM4,MYBL2,MYC,NDC80,NUSAP1,PCLAF,RRM2,SOAT2,STMN1,TNFSF9,TOP2A,TYMS,UHRF1,VCAM1
RAF1	Activated	2.42	2.34E-06	AKAP12,CD74,CDC25A,CDC6,CHN1,CXCR5,DUSP4,ERBB2,ESR1,IL10,MKI67,MYBL2,MYC,RGS1,RRM2,SH2D1A,SPRY2
TNFSF12	Activated	2.39	0.0229	CCR5,CXCR5,DLL1,JAG2,MEF2B,VCAM1
PI3K(family)	Activated	2.39	0.0202	BIRC5,CDH1,ESR1,HMOX1,IL10,PDGFB
EGR1	Activated	2.39	0.036	CCL4,CDC20,CDH1,CHGB,DUSP4,HMOX1,MYC,VCAM1
TRAF2	Activated	2.24	0.0276	AURKB,CCNB1,CDK1,CR2,PLK1
ATF6	Activated	2.24	0.00742	BUB1,CDKN3,HMMR,TROAP,UBE2C
OGT	Activated	2.24	0.000211	BIRC5,CDH1,FOXM1,MYC,NEK2,PLK1
OSM	Activated	2.24	0.00771	ATP9A,C1R,CA6,CDH1,CHI3L2,COL6A1,CXCL13,DENND5A,EPHA1,HMOX1,IL10,MYC,OSM,PLLP,RARG,TLR3,TNFRSF11A,TOP2A,TPX2,VCAM1
CCNE1	Activated	2.22	0.00017	CCNA2,CCNB1,CDC6,MCM2,MCM4
IFNB1	Activated	2.19	0.00531	CCL4,CCR5,CD38,DKK3,DLL1,IL10,MCM10,MYC,PDCD1,PMAIP1,SPRY2,TLR3,TNFRSF4
MYD88	Activated	2.19	0.0448	CCL4,CD38,CISH,CXCL13,HMOX1,IL10,MCM2,MKI67,MYC,VCAM1
MOG	Activated	2.18	0.000932	CCR5,CTLA4,IL10,MKI67,PDCD1,VCAM1
NFkB (complex)	Activated	2.02	0.00564	ADGRE1,BIRC5,C1R,CCL4,CCNB1,CCNB2,CCR7,CD74,CDH1,E2F7,ERBB2,FABP5,GZMK,HLA-DMB,HMOX1,IL10,IL2RA,MYC,PLK1,PMAIP1,TNFRSF4,TNFRSF9,VCAM1
Nfat (family)	Activated	2.00	0.12	DKK3,IL10,PDGFB,VCAM1
DOCK8	Activated	2.00	0.0546	CCL4,CD70,RGS1,TLR3
NELFCD	Activated	2.00	1.18E-05	CCNB2,DLGAP5,MYBL2,TOP2A
FGFR3	Activated	2.00	0.000628	CCL4,CDH1,CR2,DLL1,NOG
NELFA	Activated	2.00	0.000021	CCNB2,DLGAP5,MYBL2,TOP2A
Hbb-b2	Activated	2.00	0.00134	CCR5,CD74,CXCR5,CXCR6,TF
FLT1	Activated	2.00	0.0754	BCAT1,BIRC5,HMOX1,LIMS1
NELFE	Activated	2.00	0.000021	CCNB2,DLGAP5,MYBL2,TOP2A
Sod	Inhibited	-2.00	9.37E-06	CCL4,HLA-DRA,HMOX1,PMAIP1,VCAM1
HCAR2	Inhibited	-2.00	0.000347	KIF14,KIF20A,PLK1,PLK4
TNFSF13	Inhibited	-2.00	0.00134	CDC6,MCM2,MCM4,MYC,POLE2
TOB1	Inhibited	-2.00	0.016	CCNA2,CDT1,HJURP,RPRM
ATF3	Inhibited	-2.00	0.113	AURKB,CDK1,NEK2,PLK1
E2F6	Inhibited	-2.00	0.00004	CDC25A,CDC6,GINS2,MCM2,MYC,PCLAF,RAD51AP1,RRM2
TP63	Inhibited	-2.03	0.0063	CCNA2,CCNB1,CDH1,CDK1,DLL1,GLS2,JAG2,KIF23,KRT1,MCM10,MYC,NT5E,PMAIP1,RGS13,RUNX2,UPK2
Rb	Inhibited	-2.04	3.15E-10	AURKB,BIRC5,CCNA2,CDC6,CDH1,CDK1,CDT1,ECT2,FOXM1,MYBL2,MYC,PLK1,TOP2A,TYMS
IL31	Inhibited	-2.20	3.39E-05	CCNB1,CDK1,CISH,MCM4,SOCS2

CAT	Inhibited	-2.20	0.00398	*HLA-DRA,HMOX1,IL2RA,PMAIP1,VCAM1*
CREM	Inhibited	-2.21	0.207	*CCNB1,DUSP4,HLA-DRA,IRS2,TSHR*
CTLA4	Inhibited	-2.23	0.000505	*CCNA2,IL10,IL2RA,MKI67,PDCD1,TOP2A,TPX2*
TP73	Inhibited	-2.30	7.53E-05	*ADAM23,BIRC5,C1R,CCNB1,CDH1,CDK1,CNN3,DAB1,EDA,FOXJ1,HIST1H2BH,JAG2,KCNQ1,LZTS1,PDGFB,PMAIP1,PROCR,RPRM,STMN1*
ESR2	Inhibited	-2.31	0.00156	*ACER1,AKAP12,ANK1,ASB2,BCAT1,BIRC5,CCNA2,CDC25A,CDC42BPA,CDH1,ESR1,FOXM1,MKI67,MYC,PMAIP1,PTGDS,RUNX2,SOCS2,SUSD4*
RBL1	Inhibited	-2.32	4.21E-09	*AURKB,CCNA2,CDC25A,CDC6,CDK1,MCM10,MCM2,MYBL2,MYC,NEK2,PLK1,RRM2,TYMS*
BNIP3L	Inhibited	-2.33	1.75E-06	*ANK1,CCNA2,CDKN3,CENPF,KIF11,MYBL2,NUF2,RRM2,TOP2A*
DYRK1A	Inhibited	-2.44	4.26E-06	*CCNB1,CDC6,CDK1,MCM4,MYBL2,UBE2C*
WNT3A	Inhibited	-2.57	0.0367	*AXIN2,C2orf40,CLEC11A,FHL2,IRS2,LEF1,MYC,NGFR,RUNX2,VCAM1*
ZFP36	Inhibited	-2.78	1.79E-06	*CDC6,IL10,KIF11,KIF14,NEK2,NHSL2,NUF2,OSM,PBK,TOP2A,VCAM1*
RBL2	Inhibited	-2.89	2.78E-11	*AURKB,BUB1,BUB1B,CCNA2,CCNB1,CCNB2,CDC25A,CDC6,CDK1,KIFC1,MYBL2,MYC,NEK2,PLK1,RRM2,STMN1,TYMS*
TP53	Inhibited	-2.90	9.2E-23	*ACTN1,AKAP12,ANK1,ANLN,ASF1B,ASPM,AURKB,AXIN2,BIRC5,BUB1,BUB1B,CCL4,CCNA2,CCNB1,CCNB2,CD70,CDC20,CDC25A,CDC42BPA,CDC6,CDH1,CDK1,CDKN3,CDT1,CENPF,CEP55,CNN3,CRACR2B,DDIAS,DKK3,DLGAP5,DPP4,DUSP4,EPS8,ERBB2,ESPL1,ESR1,EXO1,FAM83D,FHL2,FOXM1,GAL3ST4,GLS2,HJURP,HMMR,HMOX1,IL10,IL2RA,IL5RA,KIF23,KIFC1,MAPK12,MCM2,MCM4,MELK,MKI67,MYBL2,MYC,NCAPG,NDC80,NEK2,NUSAP1,PACSIN1,PBK,PCLAF,PDGFB,PDIA5,PLK1,PMAIP1,PODXL,POLE2,PRKN,PTGDS,PTPRO,RAD51AP1,RGS13,RPRM,RRM2,RUNX2,SALL2,SH2D1A,SOCS2,STMN1,TCEA3,TNFRSF9,TNFSF9,TOP2A,TPX2,TSPAN6,TTK,TYMS,TYMSOS,UBE2C,UHRF1,UNC5B*
RB1	Inhibited	-2.95	5.33E-07	*ASF1B,AURKB,BIRC5,CCNA2,CCNB1,CDC25A,CDC6,CDCA5,CDH1,CDK1,CDT1,E2F3,EPS8,MCM10,MCM2,MCM4,MELK,MYBL2,MYC,PCLAF,PLK1,RAD51AP1,RRM2,RYR1,TYMS*
SMARCB1	Inhibited	-2.97	2.48E-06	*CCNA2,CDC6,CDH1,CDK1,CDT1,ERBB2,KIF11,KIF23,MCM10,MCM2,MCM4,MYBL2,MYC,PLK4,RAD51AP1*
CDKN1A	Inhibited	-3.09	2.79E-25	*ANLN,ASPM,AURKB,BIRC5,BUB1,BUB1B,CCNA2,CCNB1,CDC20,CDC25A,CDC6,CDK1,CDKN3,CENPF,CEP55,DLGAP5,DTL,EXO1,FOXM1,HJURP,KIF20A,KIF2C,MCM2,MCM4,MKI67,MYBL2,MYC,NUSAP1,PBK,PCLAF,PLK1,STMN1,TOP2A,TPX2,TTK,TYMS,UBE2C,UHRF1,ZNF629*
CDKN2A	Inhibited	-3.30	3.58E-12	*ASF1B,AURKB,BIRC5,BUB1B,CCL4,CCNA2,CCNB1,CCR7,CDC25A,CDCA5,CDK1,CXCL13,CXCR5,E2F3,EPS8,FHL2,IL5RA,KIFC1,LEF1,MCM4,MELK,MKI67,MS4A1,MYBL2,MYC,PLK1,PMAIP1,RAD51AP1,RRM2*
KDM5B	Inhibited	-3.48	4.43E-10	*BUB1B,CCNB1,CDCA3,CDK1,DLGAP5,ECT2,ERBB2,ESR1,FABP5,HACD1,HMMR,KIF2C,MCM2,NDC80,NEFL,PBK,TOP2A,TTK*
Irgm1	Inhibited	-3.73	9.71E-14	*AURKB,BUB1,CCNA2,CCNB1,CCNB2,CDCA3,DTL,GINS1,KIF20A,MKI67,NCAPG,NEK2,RRM2,UBE2C*
TCF3	Inhibited	-3.80	5.51E-22	*ANLN,ASF1B,AXIN2,BACH2,BIRC5,BUB1,CCNA2,CCNB1,CCNB2,CDC20,CDCA3,CDH1,CDK1,CDKN3,CEP55,CR2,ECT2,HMMR,IL2RA,KIF11,KIF2C,KIF4A,LEF1,MAL,MEF2B,MKI67,MYC,NCAPG,NDC80,NEK2,NUF2,NUSAP1,PLK4,RRM2,SERINC5,SPC24,TF,TOP2A,TOX,TTK,TYMS,ZP1,ZP3*
NUPR1	Inhibited	-4.32	1.67E-12	*AKAP12,ANK1,ASIC1,ASPM,ATP6V0A1,BUB1,BUB1B,CCNA2,CCNB2,CDCA2,CDCA3,CKAP2L,EPGN,ESPL1,ETV1,EXO1,FAM111B,FHL2,GINS1,HJURP,IRS2,KIF11,KIF20A,KIF23,KIF2C,KIFC1,KNL1,MCM10,MKI67,MYC,NGFR,PLK1,POLE2,POLQ,SHCBP1,SLC16A10,SPATS2L,SPC24,UNC5B*

Supplemental Figure 7. Ingenuity pathway analysis for RNA sequence data from nivolumab-bound vs. unbound CD8 T cells.

Upstream Regulator Analysis in QIAGEN's Ingenuity Pathway Analysis (IPA, QIAGEN Redwood City, CA, USA; www.qiagen.com/ingenuityIPA) was performed to predict the activation or inhibition of transcription factors (TFs) based on relationships with genes in the dataset and the direction of changes in expression in nivolumab-bound vs. unbound CD8 T cells (IgG4$^+$ vs. IgG4$^-$ CD8 T cells). The z-score for each TF indicated that strongest predicted activation corresponds to z-score ≥ 2, and the strongest predicted inhibition corresponds to z-score ≤ −2. Significance was calculated using the right-tailed Fisher's exact test.

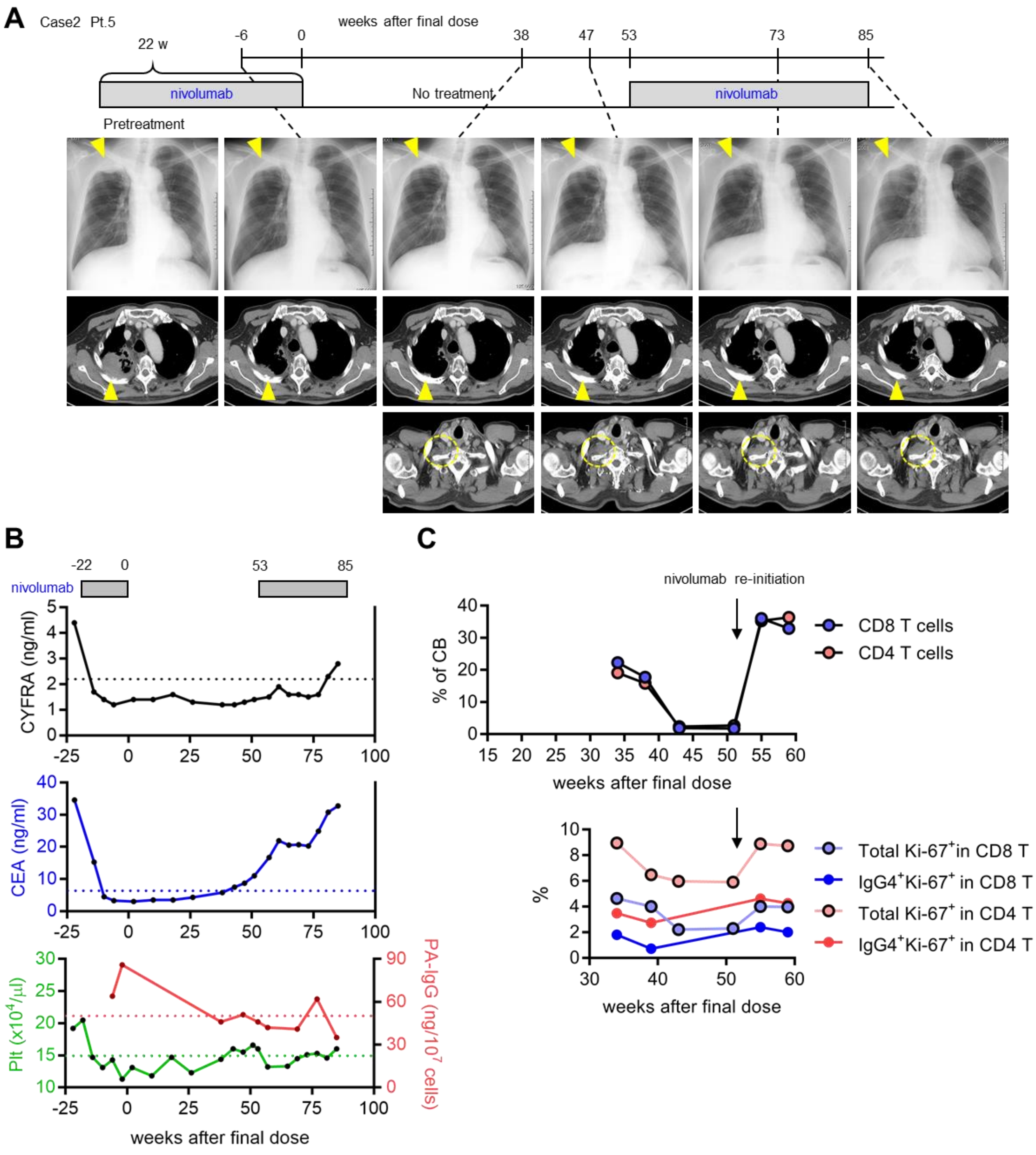

Supplemental Figure 8. A case in which monitoring of nivolumab binding was important for evaluating residual efficacy and toxicity.

(**A**) Case 2 (Pt. 5) discontinued nivolumab treatment after 22 weeks due to thrombocytopenia and fever. Chest X-ray and CT images were acquired pretreatment (left), 16 weeks after initiation of nivolumab (second column), and 38, 47, 73, and 85 weeks after the final dose (third to sixth

columns). Yellow arrow and yellow dotted circle show tumor in the right upper lung. (**B**) Follow-up data: CYFRA (ng/ml), CEA (ng/ml), platelet counts (× 10^4/μl), and platelet-associated IgG (ng/10^7 cells) at the indicated time points. (**C**) Percent of complete nivolumab binding in CD8 and CD4 T cells (upper) and Ki-67 positivity in total and nivolumab-bound CD8 and CD4 T cells (lower). Black arrow shows the time point of re-initiation of nivolumab (53 weeks after final dose).

Supplementary Information

Clinical implications of monitoring nivolumab immunokinetics in previously treated non–small cell lung cancer patients

Akio Osa, Takeshi Uenami, Shohei Koyama, Kosuke Fujimoto, Daisuke Okuzaki, Takayuki Takimoto, Haruhiko Hirata, Yukihiro Yano, Soichiro Yokota, Yuhei Kinehara, Yujiro Naito, Tomoyuki Otsuka, Masaki Kanazu, Muneyoshi Kuroyama, Masanari Hamaguchi, Taro Koba, Yu Futami, Mikako Ishijima, Yasuhiko Suga, Yuki Akazawa, Hirotomo Machiyama, Kota Iwahori, Hyota Takamatsu, Izumi Nagatomo, Yoshito Takeda, Hiroshi Kida, Esra A. Akbay, Peter S. Hammerman, Kwok-kin Wong, Glenn Dranoff, Masahide Mori, Takashi Kijima, Atsushi Kumanogoh

Supplementary Methods

Patient eligibility criteria

Key inclusion criteria for patient enrollment are listed below.

1. Pathologically confirmed non–small cell lung cancer.
2. Stage VI or recurrent diseases (without any indications for operation or radiotherapy) with peripheral blood and/or pleural effusion that can be taken.
3. Have measurable lesion in lung using the RECIST ver1.1 (Response Evaluation Criteria In Solid Tumors) criteria.
4. At least 20 years old at the time of informed consent.
5. Have adequate organ function within 14 days before enrollment.
6. Estimated life expectancy of at least 12 weeks.
7. Have signed an informed consent document.

Key exclusion criteria for patient enrollment are listed below.

1. Interstitial pneumonia or pulmonary fibrosis on chest X-ray.
2. Clinically significant drug allergy.
3. Presence of other active malignancy.
4. Pericardial effusion, pleural effusion, or ascites in need of drainage.
5. Positive serum hepatitis B virus antigen.
6. Severe infection, cardiac diseases, hypertension, or other severe complication.

RNA sequence data analysis

The Illumina Casava1.8.2 software was used for base calling. Sequenced reads were mapped to the human reference genome sequences (hg19) using TopHat v2.0.13 in combination with Bowtie2 ver. 2.2.3 and SAMtools ver. 0.1.19. Fragments per kilobase of exon per million mapped fragments (FPKM) was calculated using Cuffnorm version 2.2.1. After calculating differential expression of genes for IgG4-negative vs. IgG4-positive CD8 T cells in Pt.1s–5s, 485 genes were up- or down-regulated > 2.0-fold in IgG4-negative vs. IgG4-positive CD8 T cells (see Supplemental Figure 5 and Supplemental Table 3). Fold changes were evaluated using the Mann-Whitney U test ($p < 0.05$) using the Subio Platform and Subio Basic Plug-in (v1.20; Subio Inc., Amami, Japan). The raw data have been deposited in the NCBI Gene Expression Omnibus database (GEO accession GSE100860).

To determine whether cell cycle–related gene sets of interest were statistically enriched among up- and downregulated genes, we analyzed our non-redundant list of genes using GSEA 3.0. The gene sets used in this study (e.g., 'C5 GO biological process collection') were obtained from the Broad Molecular Signatures Database (MSigDB).

The Ingenuity Pathway Analysis (IPA) regulation z-score algorithm (Ingenuity Systems, www.ingenuity.com) was used to identify upstream pathways that were significantly differentially regulated in IgG4-positive vs. IgG4-negative CD8 T cells. We considered only functions with z-score > 2. P-values were calculated using the right-tailed Fisher's exact test to determining the probability that each functional network or pathway was assigned to that data due to chance alone. To define the similarity of differentially expressed genes, we used the Systems Biology analysis tool NextBio. Gene overlap p-values by NextBio were calculated using Fisher's exact test.

Supplementary Material

Antibody list

Antigen	Clone name	Fluorescence	Manufacturer
CD3e	OKT3<br>UCHT1	BV 510<br>Pacific Blue	BioLegend<br>BD Biosciences
CD8a	HIT8a<br>HIT8a<br>RPA-T8	PE Cy7<br>APC<br>BV 510	BioLegend<br>BioLegend<br>BioLegend
CD4	RPA-T4<br>OKT4	APC Cy7<br>PE Cy7	BioLegend<br>BioLegend
CCR7	G043H7	PerCP Cy5.5	BioLegend
CD45RA	HI100	FITC	BioLegend
CD19	HIB19	FITC	BioLegend
CD56	HCD56	FITC	BioLegend
TCRab	IP26	PerCP Cy5.5	BioLegend
IgG4 Fc / Isotype	HP6025/ ab81200	PE	Abcam
Tbet / Isotype	4B10/ MOPC-21	APC	BioLegend
Ki-67 / Isotype	Ki-67/ MOPC-21	BV 421	BioLegend
CTLA-4 / Isotype	L3D10 / MOPC-21	APC	BioLegend
CD73	AD2 / MOPC-21	BV 421	BioLegend
CXCR6	K041E5/ MOPC-21	BV 421	BioLegend
PD-1 / Isotype	EH12.1/ MOPC-12	PE Cy7	BD Biosciences

Supplementary Information

Clinical implications of monitoring nivolumab immunokinetics in previously treated non–small cell lung cancer patients

Akio Osa, Takeshi Uenami, Shohei Koyama, Kosuke Fujimoto, Daisuke Okuzaki, Takayuki Takimoto, Haruhiko Hirata, Yukihiro Yano, Soichiro Yokota, Yuhei Kinehara, Yujiro Naito, Tomoyuki Otsuka, Masaki Kanazu, Muneyoshi Kuroyama, Masanari Hamaguchi, Taro Koba, Yu Futami, Mikako Ishijima, Yasuhiko Suga, Yuki Akazawa, Hirotomo Machiyama, Kota Iwahori, Hyota Takamatsu, Izumi Nagatomo, Yoshito Takeda, Hiroshi Kida, Esra A. Akbay, Peter S. Hammerman, Kwok-kin Wong, Glenn Dranoff, Masahide Mori, Takashi Kijima, Atsushi Kumanogoh

Supplemental Tables 1–3

Supplemental Table 1

Follow-up study design and schedule after nivolumab discontinuation

Follow-up study design

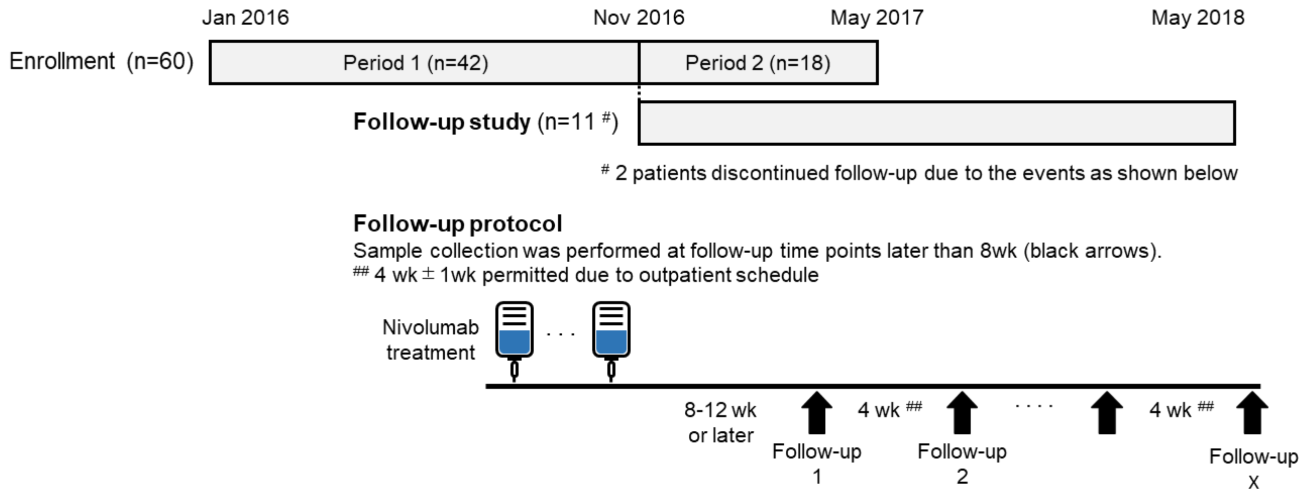

Follow-up schedule

Patient No	Started period and dose of nivolumab	Follow-up 1	Follow-up 2	Follow-up 3	Follow-up 4	Follow-up 5	Follow-up 6	Follow-up 7
Pt.5	Period 1 12 doses	34w	38w	43w				
Pt.6	Period 1 5 doses	24w	29w	32w				
Pt.7	Period 1 2 doses	26w	30w					
Pt.8	Period 1 15 doses	8w	12w	16w	19w	24w		
Pt.9	Period 1 13 doses	8w	#1	15w	20w	24w	28w	33w
Pt.10	Period 1 7 doses	10w	14w	18w	22w	26w	30w	34w
Pt.11	Period 1 5 doses	10w	14w	#2				
Pt.12	Period 1 3 doses	8w	11w	#3				

Patient No	Started period and dose of nivolumab	Follow-up 1	Follow-up 2	Follow-up 3	Follow-up 4	Follow-up 5	Follow-up 6	Follow-up 7
Pt.13	Period 2 8 doses	12w	16w	19w	23w	28w		
Pt.14	Period 2 3 doses	10w	14w	#4	#4	#4	30w	
Pt.15	Period 2 14 doses	8w	12w	16w	20w	24w	28w	

#1 Sample collection failed
#2 Nivolumab was discontinued due to irAE, and best supportive care was chosen
#3 Nivolumab was restarted,
#4 Sample collection failed due to follow-up by different department during this period

Supplemental Table 2

Patient characteristics

Case	Age, Gender	Histology	Prior regimens	Duration of nivolumab treatment
Pt.1	62 M	Adeno	1	2 doses x 2 wk
Pt.2	67 M	Adeno	1	10 doses x 2 wk
Pt.3	67 M	Adeno	2	8 doses x 2 wk
Pt.4	62 M	Squamous	5	1 dose x 2 wk
**Case**	**Age, Gender**	**Histology**	**Prior regimens**	**Duration of nivolumab treatment**
Pt.5	67 M	Squamous	2	12 doses x 2 wk
Pt.6	72 M	Adeno	3	5 doses x 2 wk
Pt.7	68 M	Squamous	1	2 doses x 2 wk
Pt.8	76 M	Adeno	8	15 doses x 2 wk
Pt.9	69 M	Squamous	1	13 doses x 2 wk
Pt.10	68 F	Adeno	5	7 doses x 2 wk
Pt.11	76 M	Adeno	2	5 doses x 2 wk
Pt.12	87 M	Squamous	1	3 doses x 2 wk
Pt.13	67 M	Squamous	2	8 doses x 2 wk
Pt.14	68 M	Adeno	1	3 doses x 2 wk
Pt.15	75 M	Adeno	5	14 doses x 2 wk

Case	Age, Gender	Histology	Prior regimens	Duration of nivolumab treatment
1s	66 M	Adeno	1	> 10 doses x 2 wk
2s Pt.13	67 M	Squamous	1	8 doses x 2 wk
3s	81 M	Squamous	2	5 doses x 2 wk
4s	61 F	Adeno	5	3 doses x 2 wk
5s	68 M	Adeno	8	5 doses x 2 wk

Supplemental Table 3

Significantly differentially regulated genes in the IgG4[+] nivolumab-bound population vs. the IgG4[-] nivolumab-unbound population

Up-regulated				Down-regulated		
Gene name	fold change ratio	p-value		Gene name	fold change ratio	p-value
TMEM155	58.10	0.009		*SNORA7B*	0.03	0.034
SNORA16B	54.72	0.034		*ADAM23*	0.07	0.026
PDCD1	27.02	0.009		*ANKRD36BP2*	0.07	0.005
PACSIN1	12.87	0.009		*SCARNA16*	0.08	0.016
PP12613	11.66	0.008		*EDN3*	0.08	0.028
ETV1	10.69	0.009		*NEFL*	0.08	0.028
TNFRSF9	8.05	0.009		*MIR1248*	0.08	0.028
CCNB2	7.84	0.009		*RMRP*	0.09	0.047
DLGAP5	7.80	0.028		*SUSD4*	0.09	0.016
HJURP	7.56	0.009		*CA6*	0.10	0.009
KIAA0101	7.00	0.009		*MXRA8*	0.11	0.047
MND1	6.60	0.047		*SFRP5*	0.11	0.005
TYMS	6.39	0.047		*AEBP1*	0.12	0.009
FAM111B	6.35	0.016		*PLXNA4*	0.12	0.005
SGPP2	6.21	0.016		*TNFRSF11A*	0.12	0.016
CXCR5	6.04	0.009		*TMPRSS11E*	0.12	0.005
KIF20A	5.98	0.008		*PCSK5*	0.12	0.009
CXCL13	5.91	0.034		*SLC1A7*	0.13	0.028
UBE2C	5.80	0.009		*EDA*	0.13	0.009
HMMR	5.66	0.028		*ISM1*	0.13	0.016
HIST1H2BH	5.63	0.016		*TCEA3*	0.14	0.028
GZMK	5.61	0.009		*FLJ21408*	0.15	0.034
TOX2	5.54	0.016		*ADTRP*	0.16	0.026
ARHGAP19-SLIT1	5.43	0.041		*EFHC2*	0.16	0.009
CDC20	5.39	0.009		*TM4SF19*	0.16	0.016
CHN1	5.38	0.009		*KANK1*	0.16	0.009
TMEM171	5.33	0.047		*EPHA1*	0.16	0.009
MYBL2	5.33	0.009		*RFPL2*	0.17	0.016
DTL	5.29	0.009		*AK5*	0.17	0.016

IL26	5.18	0.024		*LOC285965*	0.17	0.009
DEPDC1	5.16	0.009		*CRB3*	0.17	0.009
EXO1	5.12	0.009		*MAGI2-AS3*	0.17	0.008
CDCA5	5.04	0.016		*PLLP*	0.18	0.005
NCAPG	5.02	0.016		*MB21D2*	0.18	0.009
IL10	4.95	0.009		*HDGFRP3*	0.18	0.009
CXCR6	4.94	0.009		*CCL27*	0.18	0.019
TOP2A	4.71	0.009		*KLF7*	0.19	0.009
LOC100131234	4.70	0.047		*SCML1*	0.19	0.009
CEP55	4.64	0.009		*CR2*	0.19	0.005
CDC6	4.64	0.028		*SLC22A17*	0.19	0.009
MKI67	4.60	0.047		*EFHA2*	0.19	0.016
ASB2	4.57	0.009		*TNFRSF4*	0.19	0.028
NEK2	4.54	0.009		*CNN3*	0.20	0.016
SPRY2	4.50	0.047		*LOC729041*	0.20	0.009
C18orf56	4.48	0.005		*LINC00092*	0.20	0.009
CCR5	4.47	0.016		*C2orf40*	0.20	0.028
UHRF1	4.44	0.028		*PRSS30P*	0.20	0.009
BIRC5	4.39	0.028		*CLEC11A*	0.20	0.028
SLCO4A1	4.36	0.009		*IL2RA*	0.21	0.016
SHCBP1	4.28	0.028		*MDS2*	0.21	0.016
DUSP4	4.21	0.016		*LOC389493*	0.21	0.019
MCM10	4.20	0.028		*TGIF2-C20ORF24*	0.22	0.047
SPC24	4.16	0.016		*SLC16A10*	0.22	0.009
KIFC1	4.15	0.047		*FAM63A*	0.22	0.009
KIF23	4.15	0.016		*TXNDC3*	0.22	0.047
LOC728978	4.14	0.034		*C17orf67*	0.22	0.009
PBK	4.12	0.016		*GPC2*	0.23	0.013
VCAM1	4.12	0.009		*MAL*	0.23	0.009
RGS13	4.11	0.028		*ANK1*	0.23	0.047
CCL23	4.04	0.034		*STXBP1*	0.23	0.009
FBXO43	4.02	0.007		*RAB43*	0.24	0.009
MS4A1	3.94	0.047		*P2RY14*	0.24	0.028
RBM20	3.90	0.013		*SHC2*	0.24	0.019
TROAP	3.90	0.016		*ACTN1*	0.24	0.047

KIF15	3.85	0.016		*EDAR*	0.24	0.034
RRM2	3.80	0.047		*TCEAL2*	0.24	0.009
CHI3L2	3.79	0.009		*CYP2J2*	0.25	0.013
AURKB	3.76	0.028		*NOG*	0.25	0.005
ADAM29	3.74	0.005		*EEF1DP3*	0.25	0.047
KIF4A	3.71	0.028		*NT5E*	0.25	0.009
ANLN	3.70	0.028		*PRRT1*	0.25	0.008
E2F7	3.68	0.015		*NPAS2*	0.25	0.009
CDCA2	3.64	0.028		*PTGDS*	0.25	0.047
ASPM	3.57	0.016		*SOX8*	0.25	0.008
PLK1	3.56	0.047		*KRT72*	0.25	0.047
TTK	3.56	0.016		*KRT1*	0.25	0.007
CTLA4	3.47	0.009		*CDH1*	0.26	0.047
CDT1	3.45	0.016		*ZP1*	0.26	0.028
CKAP2L	3.42	0.028		*ZP3*	0.26	0.016
DSCC1	3.42	0.016		*EFHD1*	0.26	0.041
PHEX	3.38	0.005		*IL5RA*	0.26	0.028
CDC25A	3.38	0.028		*CNKSR2*	0.26	0.047
KIF14	3.34	0.034		*FOXJ1*	0.27	0.026
ZWINT	3.34	0.016		*NGFR*	0.27	0.015
TNFSF9	3.34	0.028		*CYP4F12*	0.27	0.024
CD38	3.31	0.028		*GAL3ST4*	0.27	0.028
TSHR	3.30	0.028		*C1orf177*	0.27	0.016
ADAM28	3.28	0.009		*HPCAL4*	0.27	0.047
PKMYT1	3.27	0.009		*DNAH10*	0.27	0.045
DEPDC1B	3.21	0.028		*PCSK4*	0.27	0.016
FABP5	3.21	0.009		*SNED1*	0.28	0.028
KIF11	3.21	0.016		*VIPR1*	0.28	0.009
CENPF	3.20	0.047		*BAIAP2L1*	0.28	0.009
UPK2	3.20	0.034		*ACVR2A*	0.28	0.009
KIF2C	3.17	0.028		*BACH2*	0.28	0.009
REREP3	3.15	0.016		*AMN*	0.28	0.009
TPX2	3.13	0.028		*ZFR2*	0.28	0.045
TOX	3.12	0.009		*CBR3-AS1*	0.28	0.028
RAD51AP1	3.10	0.028		*CSNK1G2-AS1*	0.28	0.045

POLQ	3.10	0.009		*LINC00299*	0.28	0.028
CASC5	3.09	0.009		*EMR1*	0.28	0.028
DKK3	3.09	0.028		*LAPTM4B*	0.29	0.047
AGAP1	3.08	0.028		*GNLY*	0.29	0.028
CDKN3	3.07	0.009		*RASL11B*	0.29	0.016
FCRL3	3.05	0.009		*ALPK1*	0.29	0.028
CDK1	3.05	0.009		*LGALS17A*	0.29	0.041
CDCA3	3.04	0.016		*IRS2*	0.29	0.028
NUSAP1	3.03	0.016		*PTPRO*	0.29	0.009
BUB1	3.02	0.028		*CNKSR1*	0.29	0.009
GINS2	2.99	0.028		*DSEL*	0.30	0.009
GINS1	2.99	0.047		*PSPN*	0.30	0.045
KCNK5	2.98	0.028		*CISH*	0.30	0.016
ESPL1	2.96	0.047		*CD248*	0.30	0.047
BUB1B	2.94	0.028		*PROCR*	0.30	0.047
HNRPLL	2.91	0.009		*FXYD7*	0.30	0.028
AFF3	2.89	0.007		*GP5*	0.30	0.009
ATP9A	2.89	0.028		*COL9A2*	0.30	0.009
DFNB31	2.85	0.009		*RPRM*	0.30	0.041
BCAT1	2.84	0.028		*CMKLR1*	0.30	0.028
RGS1	2.83	0.009		*LOC100128252*	0.31	0.047
CCNB1	2.83	0.016		*C17orf51*	0.31	0.009
SEMA4A	2.81	0.016		*RGMB*	0.31	0.009
ST6GALNAC3	2.80	0.047		*DLL1*	0.32	0.009
PRKCDBP	2.79	0.019		*CCR7*	0.32	0.016
GJD3	2.77	0.016		*C1orf162*	0.32	0.009
PMAIP1	2.77	0.047		*HOXC5*	0.32	0.013
DTHD1	2.75	0.047		*TXK*	0.32	0.009
PTPLA	2.73	0.045		*ADAMTS10*	0.32	0.028
NUF2	2.72	0.009		*ACCN2*	0.32	0.028
C7orf61	2.71	0.016		*HS3ST3B1*	0.32	0.016
MELK	2.70	0.047		*PLAG1*	0.32	0.028
BREA2	2.69	0.009		*GRASP*	0.33	0.047
LANCL3	2.68	0.028		*BDAG1*	0.33	0.047
CD27	2.66	0.028		*ZNF516*	0.33	0.028

HLF	2.66	0.028		*PRPF40B*	0.33	0.009
C12orf42	2.61	0.028		*GREM2*	0.33	0.047
SCD	2.58	0.009		*GPR55*	0.33	0.028
CLSPN	2.57	0.047		*FRY*	0.33	0.016
RYR1	2.51	0.009		*ALS2CL*	0.33	0.009
SAPCD2	2.51	0.028		*SPTB*	0.34	0.047
TNIP3	2.50	0.016		*PARK2*	0.34	0.047
LIMS1	2.49	0.009		*LOC100506046*	0.34	0.047
LOC153469	2.48	0.013		*LINC00085*	0.34	0.016
ESR1	2.47	0.034		*GNAL*	0.34	0.047
FCGR2B	2.47	0.028		*AGPAT4-IT1*	0.34	0.047
NDC80	2.47	0.009		*RAB25*	0.34	0.019
HLA-DMA	2.46	0.009		*TRPC1*	0.34	0.009
ZBTB32	2.46	0.047		*TLR5*	0.34	0.016
CCNA2	2.46	0.047		*GDPD5*	0.34	0.016
MAPK12	2.46	0.041		*DENND5A*	0.35	0.009
LYST	2.45	0.047		*AXIN2*	0.35	0.047
CPNE2	2.43	0.009		*ACER1*	0.35	0.047
CRTAM	2.42	0.016		*C1orf228*	0.35	0.047
ITM2A	2.38	0.047		*SPINT2*	0.35	0.028
SPATS2L	2.38	0.009		*PDIA5*	0.35	0.028
TRPS1	2.35	0.016		*EPHX2*	0.35	0.009
MYO1E	2.32	0.009		*LOC100506746*	0.35	0.008
ZNF532	2.30	0.028		*IQCA1*	0.35	0.028
MCM2	2.30	0.047		*C4orf32*	0.35	0.009
GLDC	2.27	0.034		*COL6A1*	0.35	0.009
BATF3	2.25	0.016		*PFN2*	0.36	0.028
CCL4	2.24	0.016		*C2orf89*	0.36	0.009
C11orf82	2.23	0.047		*GNA11*	0.36	0.028
NINJ2	2.23	0.016		*ATP6V0A1*	0.36	0.047
AGAP7	2.23	0.047		*PLEKHG3*	0.36	0.028
CD70	2.22	0.047		*SATB1*	0.36	0.009
CYTH3	2.20	0.016		*HEMGN*	0.36	0.028
FAM83D	2.20	0.009		*DLEC1*	0.36	0.009
E2F3	2.19	0.047		*MYC*	0.36	0.009

LOC79015	2.17	0.047		*EIF4EBP3*	0.36	0.028
FOXM1	2.17	0.047		*LOC641518*	0.37	0.028
SCAMP5	2.16	0.028		*TRIM2*	0.37	0.047
MCM4	2.15	0.047		*AIM1L*	0.37	0.047
POC1A	2.15	0.028		*DAB1*	0.37	0.019
C1R	2.13	0.009		*GPR150*	0.37	0.047
PLK4	2.13	0.016		*ARHGAP32*	0.37	0.028
SH2D1A	2.13	0.009		*PIP5KL1*	0.37	0.045
ECT2	2.12	0.028		*MEF2B*	0.37	0.047
SH2B2	2.11	0.009		*PRX*	0.37	0.047
CLDND1	2.11	0.028		*ARHGEF4*	0.38	0.009
HMOX1	2.10	0.047		*EBF4*	0.38	0.047
CMC1	2.10	0.009		*FAM178B*	0.38	0.047
TIGIT	2.10	0.016		*RUNX2*	0.38	0.009
POLE2	2.10	0.047		*MTUS2*	0.38	0.019
CD74	2.10	0.009		*CHGB*	0.38	0.019
SMS	2.09	0.009		*STK32C*	0.39	0.028
HLA-DMB	2.09	0.047		*LEF1*	0.39	0.009
STMN1	2.08	0.016		*SOAT2*	0.39	0.047
HLA-DRA	2.07	0.028		*GSDMA*	0.39	0.047
AASS	2.07	0.047		*CARNS1*	0.39	0.009
EFCAB4A	2.07	0.009		*EPS8*	0.39	0.015
CIB2	2.07	0.047		*FPGT-TNNI3K*	0.39	0.047
GALM	2.05	0.047		*DPP4*	0.40	0.016
LOC643733	2.05	0.009		*RHOBTB3*	0.40	0.047
EMID1	2.05	0.019		*AGBL3*	0.40	0.009
UNC5B	2.05	0.019		*SDK2*	0.40	0.019
PDGFB	2.04	0.016		*TSPAN6*	0.40	0.028
ZNF80	2.03	0.047		*ABCB9*	0.40	0.016
PEX11A	2.03	0.047		*SOWAHC*	0.40	0.024
TLR3	2.03	0.016		*PLD6*	0.40	0.016
CDYL2	2.03	0.028		*JAG2*	0.41	0.016
WDR34	2.02	0.028		*TLE1*	0.41	0.047
FHL2	2.02	0.028		*KLHL6*	0.41	0.009
ASF1B	2.02	0.047		*FZD4*	0.41	0.047

GZMA	2.01	0.028		*PLEKHG4*	0.42	0.016
SKA1	2.00	0.016		*EPGN*	0.42	0.005
				SOCS2	0.42	0.009
				SIGLEC6	0.42	0.034
				NRIP3	0.42	0.047
				FLJ34208	0.42	0.047
				ERBB2	0.42	0.028
				ASB13	0.42	0.047
				SPIN3	0.43	0.047
				CHD7	0.43	0.016
				BAI2	0.43	0.047
				TTC8	0.43	0.047
				TTC23	0.43	0.034
				LOC100289509	0.43	0.019
				SALL2	0.43	0.028
				GOLM1	0.43	0.047
				LOC100506134	0.43	0.008
				ZNF502	0.44	0.047
				CDC42BPA	0.44	0.034
				ADD2	0.44	0.019
				LZTS1	0.44	0.034
				OSM	0.44	0.016
				MAST4	0.44	0.009
				FNIP2	0.45	0.016
				RNF24	0.45	0.009
				LONRF3	0.45	0.009
				TTC26	0.45	0.028
				ATG9B	0.45	0.028
				SMARCD3	0.45	0.016
				C14orf132	0.45	0.005
				ZNF425	0.45	0.047
				PTGER2	0.45	0.016
				LINC00511	0.46	0.041
				C1orf101	0.46	0.028
				HTR7P1	0.46	0.041

				TF	0.46	0.013
				TMEM30B	0.46	0.016
				NUDT12	0.46	0.015
				ANKK1	0.46	0.019
				KCNQ1	0.46	0.047
				NPB	0.46	0.047
				LOC100507392	0.46	0.047
				TSPAN2	0.46	0.016
				REEP6	0.46	0.047
				CSGALNACT1	0.47	0.047
				A1BG-AS1	0.47	0.028
				AKAP12	0.47	0.028
				HYI	0.47	0.047
				DIRC3	0.47	0.016
				SPON1	0.47	0.028
				FLJ35946	0.48	0.019
				MBOAT2	0.48	0.016
				RPS6KL1	0.48	0.045
				GLS2	0.48	0.028
				DCDC2B	0.48	0.047
				GPR152	0.48	0.047
				TNK1	0.48	0.047
				ACSS2	0.48	0.047
				TAS2R5	0.48	0.047
				WWC2	0.48	0.028
				DTWD2	0.48	0.016
				PLEKHA4	0.48	0.047
				CCDC13	0.48	0.015
				MSX2P1	0.49	0.009
				ZNF629	0.49	0.034
				NHSL2	0.49	0.047
				C1orf115	0.49	0.047
				SERINC5	0.49	0.028
				LOC285359	0.49	0.047
				RARG	0.49	0.028

				NEFM	0.49	0.008
				PODXL	0.50	0.028
				RNF165	0.50	0.034
				NMUR1	0.50	0.047
				RNF144A	0.50	0.009
